# Biomaterial strategies for precision regeneration of the tooth extraction socket: integrating site phenotype, pathological microenvironment, and healing sequence

**DOI:** 10.3389/fbioe.2026.1957296

**Published:** 2026-09-21

**Authors:** Hai He, Zishuai Chen, Hengxiang Liu, Zilin Qiao, Sijing Yang, Heqiang Yang, Xiaoyong Chen, Yufei Yang, Zifan Wang, Zhiqiang Li

**Affiliations:** 1 Key Laboratory of Biotechnology and Bioengineering of State Ethnic Affairs Commission, Biomedical Research Center, School of Bioengineering Northwest Minzu University, Lanzhou, China; 2 Gansu Animal Cell Technology Innovation Center, Northwest Minzu University, Lanzhou, China; 3 School of Stomatology, Key Laboratory of Oral Diseases of Gansu Province, Key Laboratory of Stomatology of State Ethnic Affairs Commission, Northwest Minzu University, Lanzhou, Gansu Province, China

**Keywords:** alveolar ridge preservation, biomaterials, clinical translation, pathological microenvironment, site phenotype, temporal regeneration, tooth extraction socket

## Abstract

Alveolar ridge remodeling after tooth extraction is not merely a process of bone defect repair; rather, it is jointly regulated by the anatomical phenotype, pathological microenvironment, surgical procedures, subsequent restorative objectives, and temporal healing sequence of the socket. Existing studies on extraction socket biomaterials are largely categorized by material composition or isolated osteogenic properties, and have yet to adequately address what the dominant regenerative barrier is at a given site, which material functions are clinically necessary, and whether increasing functional complexity translates into patient-relevant benefits. Using a critical narrative review approach, this article comprehensively analyzes the anatomical characteristics, pathological states, surgical and restorative contexts, and staged healing events of healthy and compromised extraction sockets, with particular emphasis on the impact of blood clot and regenerative space instability, biofilm and persistent inflammation, oxidative stress overload, insufficient angiogenesis, and osteogenesis–bone remodeling imbalance on material efficacy. In this review, the term “precision regeneration” refers to the selection of a minimally necessary combination of functions based on the site phenotype and its dominant barriers, rather than the indiscriminate stacking of antibacterial, anti-inflammatory, pro-angiogenic, and osteogenic functions. On this basis, we evaluate the applicability boundaries, directness of supporting evidence, and translational limitations of natural polymers, synthetic polymers, bioceramics, composite materials, and bioactive factor delivery systems across the functional dimensions of blood clot stabilization and wet-environment adhesion, space maintenance and osteoconduction, antibiofilm and immunomodulatory activity, osteogenesis–angiogenesis coupling, pathological microenvironment responsiveness, and anatomical adaptation. We further propose a decision framework of “site phenotype–dominant barrier–minimal necessary functions–material strategy–clinical net benefit,” and discuss key issues including mismatch between degradation and regeneration rates, residual materials, host cell cytotoxicity, burst release of bioactive factors, manufacturing complexity, and long-term safety. This framework is intended to drive the transition of socket preservation from empirical selection driven by material category and functional quantity toward precision regeneration strategies grounded in clinical phenotype, biological requirements, and patient outcomes.

## 1 Introduction

Although the post-extraction socket heals spontaneously through hemostasis, inflammation, tissue formation, and bone remodeling, this process does not restore the original alveolar ridge: after loss of the periodontal ligament and root, bundle bone undergoes programmed resorption, producing horizontal and vertical ridge reduction that is most pronounced at sites with a thin buccal plate (Hansson and Halldin, 2012; Tan et al., 2012; Jung et al., 2018; Stumbras et al., 2019; de Sousa Gomes et al., 2019; Avila-Ortiz et al., 2020; Couso-Queiruga et al., 2021; Guo et al., 2022; Udeabor et al., 2023). Socket preservation and guided bone regeneration therefore aim not only to promote intra-socket bone formation but also to maintain ridge contour and soft-tissue morphology, thereby securing the three-dimensional basis for subsequent implant placement (de Carvalho Formiga et al., 2019; Atieh et al., 2021; Donos et al., 2023; Madi et al., 2023; Yankov, 2023; Fok et al., 2024; Gabay et al., 2024). Accordingly, regenerative planning must consider defect morphology, bone-wall condition, soft-tissue status, and restorative objectives together.

Unlike enclosed long-bone defects, the socket is an open wound exposed to salivary flushing, oral biofilms, masticatory micromotion, and rapid soft-tissue migration; early healing therefore depends critically on stable blood-clot formation and retention, because clot disruption impairs subsequent inflammation resolution, vascular ingrowth, and osteoblast recruitment (Akinbami and Godspower, 2014; Mozzati et al., 2022; Taberner-Vallverdú et al., 2022; Khalil, 2023; Sawyer et al., 2023; Yin et al., 2025). Moreover, intra-socket bone formation and external ridge remodeling are not synchronized, so the socket represents a unique environment defined by openness and competitive soft-*versus* hard-tissue repair.

Regenerative potential also varies markedly across sites and patients. Bone-wall integrity, buccal-plate thickness, tooth position, and soft-tissue condition determine clot stability, regenerative space, and blood supply (Cardaropoli et al., 2014; Kim et al., 2021; Steigmann et al., 2022; Ghanaati et al., 2025), while diabetes, smoking, infection, and irradiation reshape the microenvironment through distinct dominant barriers (de Arruda et al., 2006; Ko et al., 2021; Kuśnierek et al., 2022; Mozzati et al., 2022; Normando et al., 2022; Yin et al., 2025). Socket preservation should therefore move from uniform material selection toward stratified, mechanism-based intervention.

Biomaterials modulate socket regeneration by stabilizing the clot, maintaining space, excluding non-osteogenic tissue, supporting cellular and vascular ingrowth, and regulating immune and microbial conditions (Leblebicioglu et al., 2013; Majzoub et al., 2019; Funayama et al., 2021; Ebenezer et al., 2022; Chisci et al., 2023; Kumar et al., 2024; Marian et al., 2024; Farazin and Darghiasi, 2025; Wang et al., 2025). Collagen, chitosan, synthetic polymers, calcium phosphates, and their composites offer complementary properties, yet most studies still evaluate materials by origin or isolated osteogenic endpoints that cannot predict long-term ridge maintenance or reduced need for secondary augmentation, and functional stacking against multiple barriers can increase cytotoxicity, loss of release control, and manufacturing complexity. Material complexity alone is therefore not a valid measure of clinical value.

The central challenge is no longer a shortage of materials but the absence of explicit principles matching material functions to site phenotype, dominant barrier, surgical and restorative context, and healing stage—particularly in high-risk sites (wall defects, thin buccal plates, infection, diabetes, smoking, irradiation, medication-related risk), where necessary and proportionate function matters more than maximal *in vitro* osteogenic activity. Although a recent review summarized socket-healing biomaterials across hemostasis, anti-infection, regeneration, factor delivery, and emerging technologies (Yin et al., 2025), existing reviews have not integrated anatomical phenotype, pathology, surgery, and restorative goals into an actionable decision framework, nor delineated the extrapolation boundaries among human socket evidence, animal socket evidence, and general bone-tissue-engineering data. This review therefore builds a precision-regeneration framework—integrating anatomical, pathological, surgical, and restorative contexts to deliver the lowest necessary complexity of material functions temporally matched to the healing sequence—and evaluates function–demand matching, evidence directness, the costs of functional integration, and translation of complexity into patient-relevant outcomes rather than cataloguing materials by origin.

## 2 Literature search and narrative synthesis methods

This critical narrative review synthesized evidence on the relationships among socket anatomical phenotype, pathological microenvironment, healing sequence, surgical and restorative context, and biomaterial functional adaptation. PubMed, MEDLINE, Web of Science Core Collection, and the Cochrane Library were searched from inception to 1 July 2026, using terms including “tooth extraction socket,” “socket healing,” “alveolar ridge preservation,” “bone regeneration,” “biomaterial,” “bone substitute,” “hydrogel,” “scaffold,” “bioceramic,” “immunomodulation,” “biofilm,” “angiogenesis,” “oxidative stress,” “diabetes,” “smoking,” “infection,” “radiotherapy,” “medication-related osteonecrosis,” and “three-dimensional printing,” combined with Boolean operators and subject headings, supplemented by forward and backward citation tracking. Only peer-reviewed English-language articles were eligible; conference abstracts, editorials, letters, errata, and unpublished data were excluded.

Studies were included for direct relevance to socket healing, alveolar ridge preservation (ARP), biomaterial design, or related clinical decision-making. Priority was given to randomized controlled trials (RCTs), prospective studies, and systematic reviews of human sockets or ARP, followed by animal socket models and alveolar or craniomaxillofacial defect studies; *in vitro* or general bone-tissue-engineering studies were included as mechanistic evidence, with their extrapolative nature indicated in the text. Studies lacking substantive relevance, duplicate publications, and entries with incomplete or unverifiable information were excluded. Case reports, technical reports, dissertations, and non-systematic reviews were not used as the primary basis for core efficacy conclusions, and systematic reviews were used mainly to identify consensus, heterogeneity, and evidence gaps rather than to replace analysis of key original studies.

The literature selection process is summarized in Figure 1. The initial search yielded 14,544 records (PubMed, 4,823; MEDLINE, 4,156; Web of Science Core Collection, 5,387; Cochrane Library, 178), plus 63 records from citation tracking. After removal of 10,127 duplicates, 4,480 records were screened by title and abstract, and 3,912 were excluded as irrelevant. Of 568 full-text articles assessed, 245 were excluded with documented reasons: 115 lacked direct relevance to the review questions; 63 provided insufficient mechanistic or outcome data; 19 were inaccessible as full text; 12 reported duplicate or overlapping cohorts; and 36 were removed during *post hoc* reference-system verification (unverifiable or non-peer-reviewed sources, dissertations, patents, and off-topic records). Ultimately, 323 studies were included in the narrative synthesis.

**FIGURE 1:**
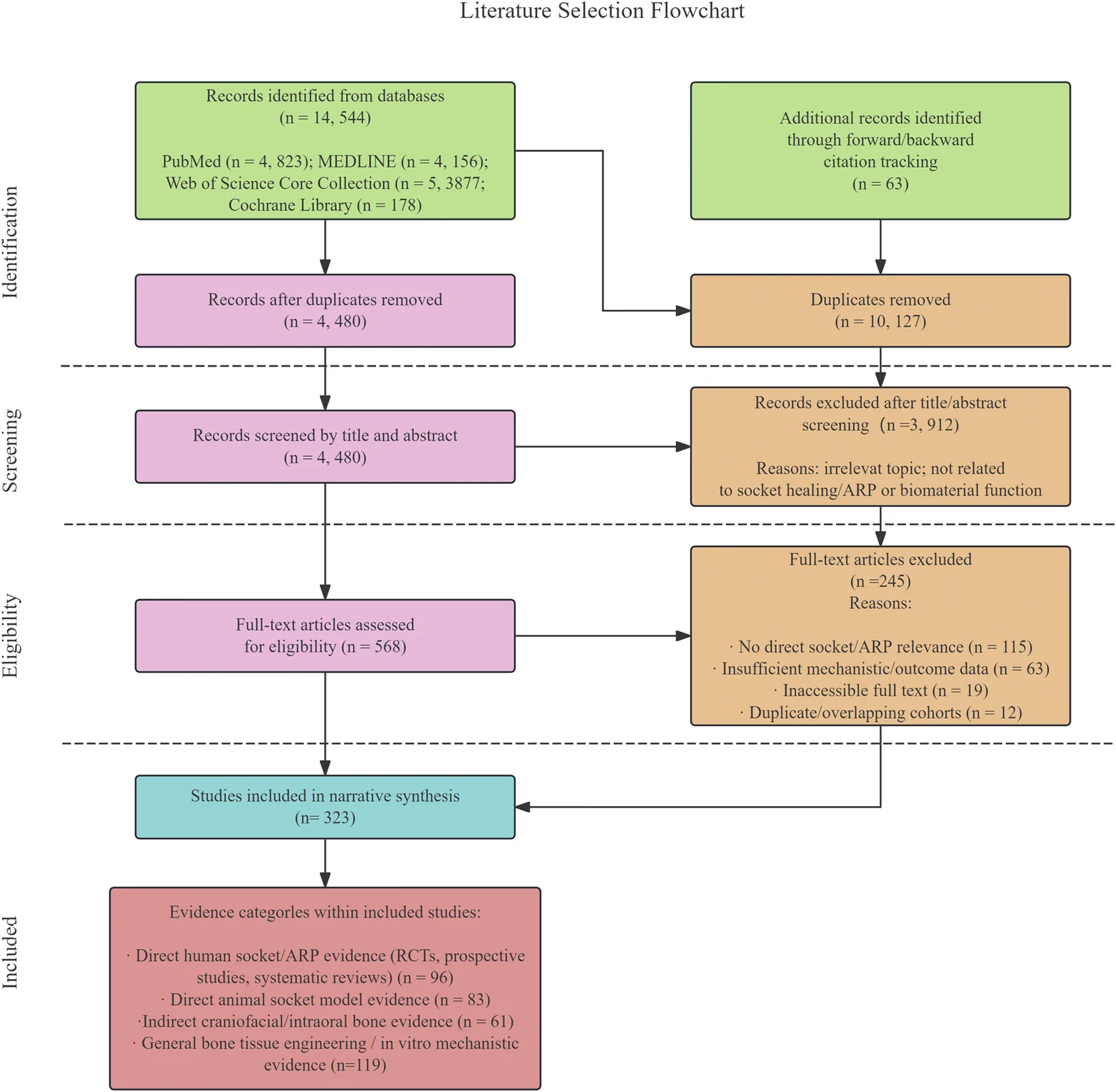
Literature selection flowchart for this critical narrative review. Records were identified through four databases (PubMed, MEDLINE, Web of Science Core Collection, Cochrane Library) and citation tracking, screened by title and abstract, and assessed in full text. Ultimately, 323 studies were included and categorized into four evidence tiers: direct human socket/ARP evidence, direct animal socket model evidence, indirect craniofacial/intraoral bone evidence, and general bone tissue engineering or *in vitro* mechanistic evidence.

Evidence was evaluated along three dimensions: directness of the source relative to the human extraction socket (direct human socket evidence; direct animal socket evidence; indirect intraoral or jawbone evidence; general bone tissue engineering evidence; *in vitro* mechanistic evidence); internal validity (study design, sample size, controls, outcome completeness, risk of bias); and distance of the endpoint from patient net benefit (mechanistic surrogates; tissue and anatomical outcomes; clinical and patient-relevant outcomes). Given high heterogeneity, no quantitative pooling or formal Grading of Recommendations Assessment, Development, and Evaluation (GRADE) rating was performed.

The synthesis combined thematic induction, mechanistic comparison, and clinical scenario analysis; extracted items included material composition and structure, mechanism of action, applicable phenotypes, experimental models, outcomes, risks, manufacturing requirements, and translational maturity. These results informed the “site phenotype–dominant barrier–minimal necessary functions–material strategy–clinical net benefit” framework. As a critical narrative review, selection and synthesis remain subject to the authors’ judgment and study heterogeneity; evidence sources and extrapolation boundaries are therefore indicated throughout.

## 3 Clinical phenotypes, biological sequence, and dominant barriers in extraction socket regeneration

This section identifies the site phenotypes and dominant regenerative barriers of extraction sockets across four dimensions—anatomical, pathological, surgical, and restorative—and further establishes a material decision pathway proceeding from the minimal necessary combination of functions and the lowest effective material complexity to clinically relevant outcomes. The overall framework is shown in Figure 2.

**FIGURE 2:**
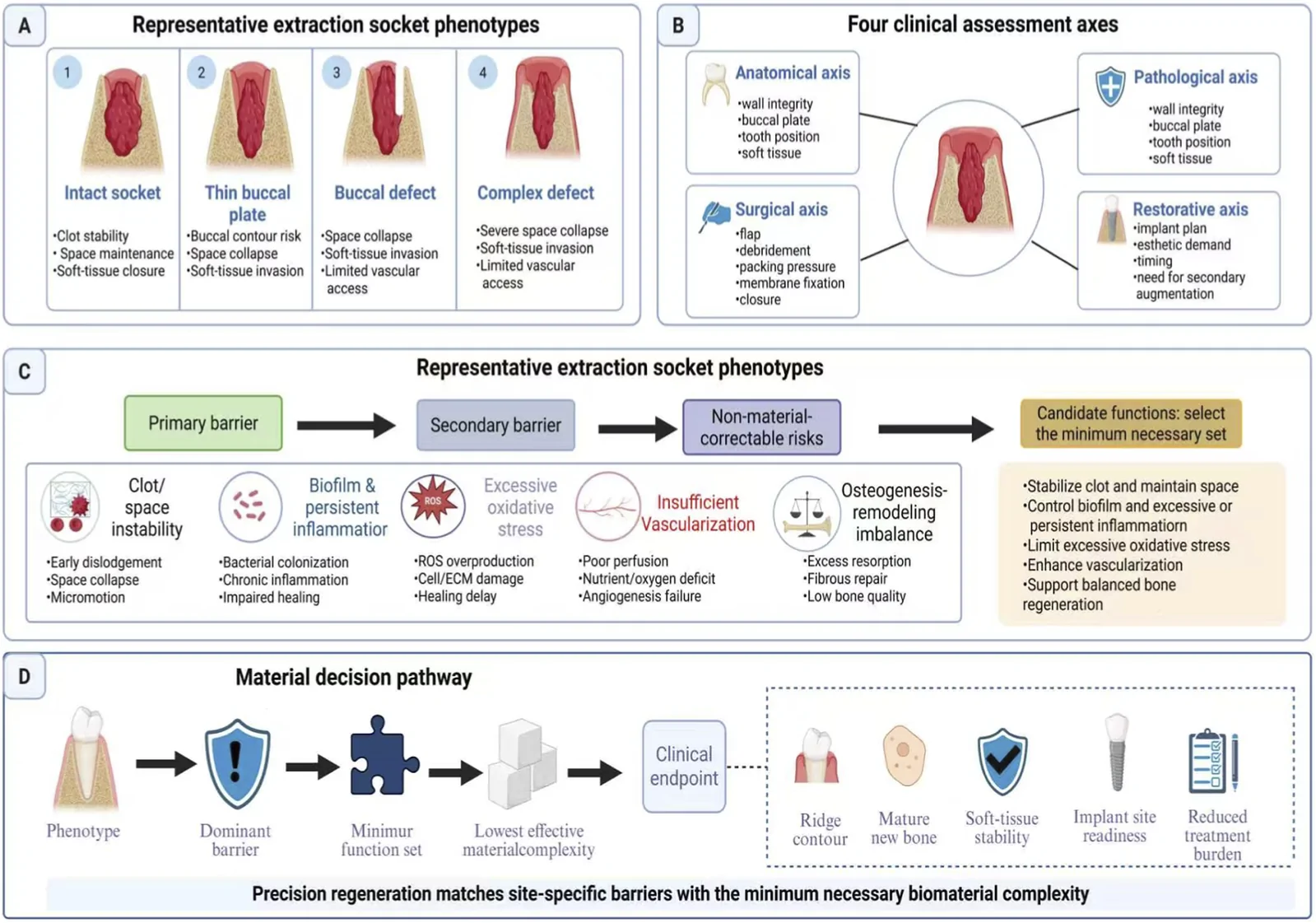
Multi-axis clinical phenotypic assessment and biomaterial decision framework for precision socket regeneration. **(A)** Anatomical phenotypes from intact four-wall sockets to complex wall defects; decreasing wall integrity increases clot displacement, space collapse, soft-tissue invasion, and vascular restriction. **(B)** Four assessment dimensions: anatomical, pathological, surgical, and restorative. **(C)** Candidate functions are combined only at the minimum necessary level after identifying primary/secondary barriers and non-correctable risks. **(D)** Decision pathway: phenotype → dominant barrier → minimal necessary functions → lowest-effective-complexity material, judged by clinically relevant outcomes.

### 3.1 Oral-specific environment and anatomical phenotypes of the extraction socket

The socket is an open wound exposed to salivary flushing, oral microorganisms, masticatory micromotion, and rapid soft-tissue migration. The blood clot serves as both hemostatic plug and provisional matrix for inflammatory recruitment, vascular ingrowth, and osteoprecursor migration, so stable clot formation and retention—rather than the osteogenic activity of an implanted material per se—largely determine baseline regenerative potential (Araújo et al., 2015; de Sousa Gomes et al., 2019; Udeabor et al., 2023; He et al., 2025; Yin et al., 2025). Bone-wall integrity is the key anatomical determinant of regenerative space, cell sources, and blood supply: intact four-wall sockets provide stable clot boundaries and abundant vascular access, whereas increasing wall defects promote clot displacement, soft-tissue invasion, and space collapse, raising requirements for barrier function, contour maintenance, and structural support (Avila-Ortiz et al., 2014; Kim et al., 2021; Steigmann et al., 2022; Elbanna et al., 2023; Ekhlasmandkermani et al., 2025).

Buccal-plate thickness and continuity further govern external ridge contour. Because much of the buccal plate is bundle bone associated with the periodontal ligament, it undergoes programmed resorption after root and ligament loss even as intra-socket bone forms (Cardaropoli et al., 2014; Schnutenhaus et al., 2018; Chang and Liu, 2022; Fok et al., 2024; Ghanaati et al., 2025); thinner plates are associated with greater horizontal and vertical loss, and baseline thickness may still influence post-ARP ridge dimensions (Matarasso et al., 2009; Avila-Ortiz et al., 2014; Spinato et al., 2014; de Carvalho Formiga et al., 2019; ElNahass et al., 2024; Madi et al., 2025). Thickness is not decisive alone, however, so fixed thresholds such as “1 mm” aid risk identification but are not absolute classification criteria (Spinato et al., 2014; Steigmann et al., 2022; ElNahass et al., 2024; Ekhlasmandkermani et al., 2025).

Tooth position and soft-tissue conditions also shape the regenerative phenotype: anterior and premolar regions have thinner buccal plates and higher esthetic demands, whereas molar sockets are larger and risk uneven material filling, central under-vascularization, and prolonged regeneration; soft-tissue thickness, keratinized-mucosa width, and closure stability affect material exposure and early tissue stability (Kim et al., 2021; Araújo et al., 2023; Minetti et al., 2024; Dai et al., 2025; Ekhlasmandkermani et al., 2025; Feng et al., 2025; Shobha et al., 2025; Tian Y. et al., 2025). Classifying sockets by alveolar bone quality or a single plate thickness therefore cannot fully reflect regenerative difficulty.

The anatomical phenotype should thus be judged across bone-wall integrity, buccal-plate thickness and continuity, tooth position, defect morphology, and soft-tissue conditions. Intact four-wall sites mainly require clot stabilization and moderate osteoconduction; thin-plate sites require enhanced contour and soft-tissue maintenance; and wall-defect sites rely more heavily on barrier function, space maintenance, material fixation, and vascularization support (Avila-Ortiz et al., 2014; Spinato et al., 2014; Kim et al., 2021; Steigmann et al., 2022; Ekhlasmandkermani et al., 2025). The purpose of anatomical phenotyping is to identify the highest-priority material functional requirements of each site, not merely to predict the amount of new bone.

### 3.2 Healing sequence, stage-specific functional requirements, and extrapolation boundaries of models

Socket healing comprises overlapping processes—hemostasis/coagulation, inflammatory clearance, tissue formation, and bone remodeling—whose onset and duration vary with species, tooth position, trauma, infection, and systemic state; description by key biological events is therefore preferable to fixed postoperative days (Araújo et al., 2015; Rahman et al., 2015; de Sousa Gomes et al., 2019; Kim et al., 2020; Udeabor et al., 2023; Yin et al., 2025).

The early phase centers on clot formation: platelet activation and the fibrin network achieve hemostasis while enriching PDGF, TGF-β, and VEGF, providing a provisional matrix for cell recruitment and early angiogenesis (Senior et al., 1986; Languino et al., 1993; Flick et al., 2004; Verma et al., 2019; de Sousa Gomes et al., 2019; Udeabor et al., 2023; Toti et al., 2025). Given the risks of salivary flushing, micromotion, fibrinolysis, and bacterial colonization, early material priorities are rapid hemostasis, wet adhesion, washout resistance, and clot protection—not immediate high-dose osteogenic factors.

Neutrophils, monocytes, and macrophages then clear necrotic tissue and debris and regulate repair *via* cytokines. Moderate, self-limiting inflammation initiates regeneration, whereas persistent pro-inflammatory signaling or uncontrolled biofilm inhibits angiogenesis and osteogenesis (McMillan, 1999; Gong et al., 2016; Raziyeva et al., 2021; Li Y. et al., 2023; Eom et al., 2025; Fonseca et al., 2025; Qi et al., 2025; Saim et al., 2025); materials should promote timely transition to a reparative milieu rather than broadly suppressing inflammation.

As granulation tissue forms, endothelial cells, MSCs, and osteogenic precursors enter the socket, and angiogenesis couples with early osteogenesis through BMP/Smad, VEGF, and Wnt/β-catenin signaling (Nishio et al., 2006; Phimphilai et al., 2006; Towler, 2007; Hu and Olsen, 2016; Komori, 2019; Song J. et al., 2024; Ouyang et al., 2025). Materials must maintain interconnected pores and a cell-permissive interface; augmenting osteogenic signals without vascularization or spatial stability yields little sustained bone.

Late remodeling converts woven to lamellar bone through osteoblast–osteocyte–osteoclast coordination and RANKL/OPG balance (Boyce and Xing, 2008; Ghalayani et al., 2014; Sadr et al., 2017; Aoyagi et al., 2018; Biguetti et al., 2019; Bolamperti et al., 2022; Fan et al., 2022; Huang et al., 2025; Kim S. et al., 2025). For grafted sockets this stage requires balancing material degradation against new-bone replacement: too-rapid degradation loses space, whereas too-slow degradation risks particle retention and delayed maturation; late endpoints should include residual-material fraction, bone maturity, and contour stability.

These stages differ across species: rodent healing at 7–21 days cannot be equated to human time points, and although dogs and minipigs better approximate human alveolar structure, differences in healing speed, loading, and microbiota persist (Araújo et al., 2015; Rahman et al., 2015; de Sousa Gomes et al., 2019; Kim et al., 2020; Udeabor et al., 2023). Stage-specific design should follow biological events—clot stabilization, inflammatory transition, angiogenesis–osteogenesis coupling, remodeling—rather than fixed postoperative days (Figure 3; Supplementary Table S1).

**FIGURE 3:**
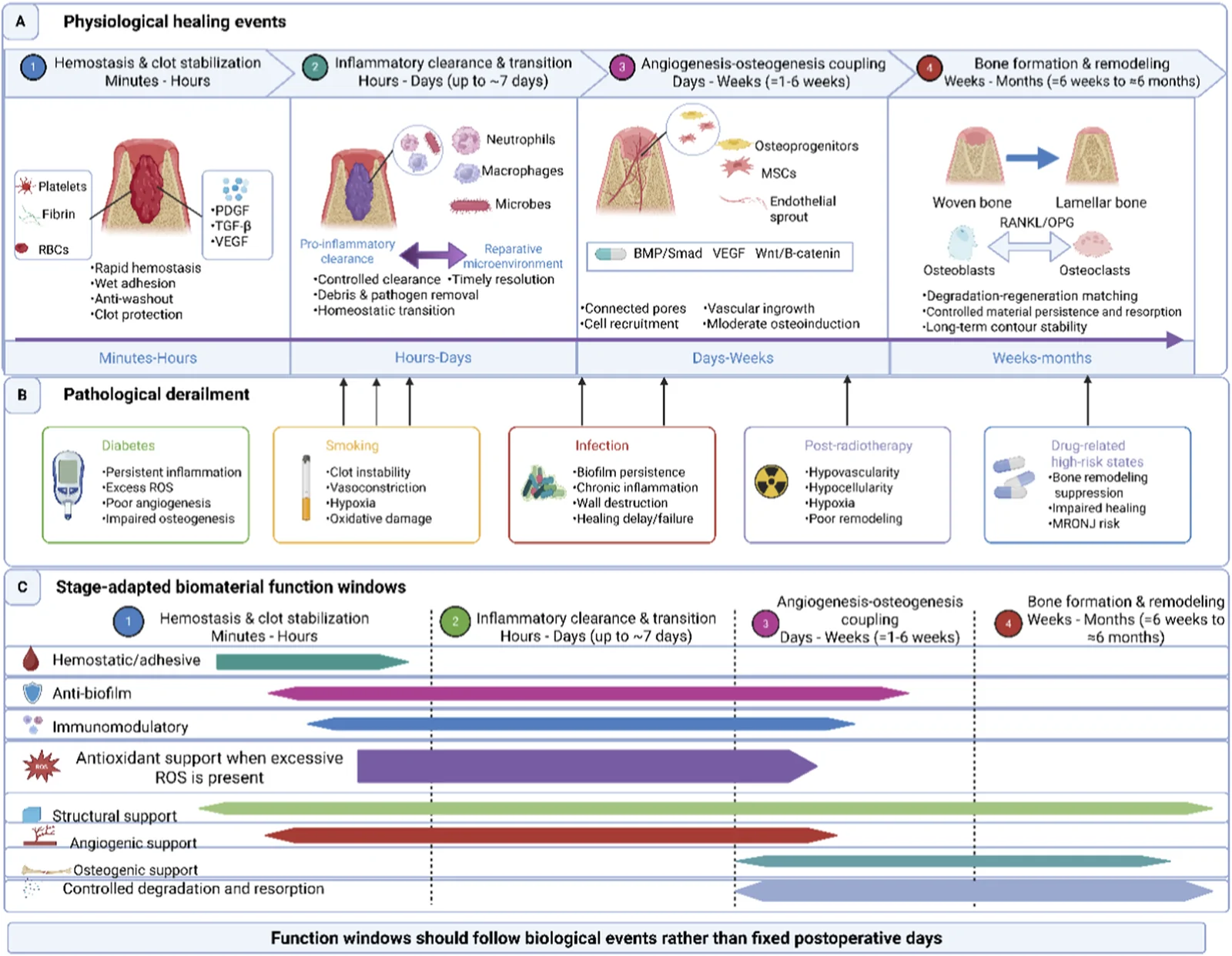
Healing sequence, pathological deviations, and stage-adapted material functional windows. **(A)** Normal healing overlaps four processes—clot stabilization, inflammatory clearance and reparative transition, angiogenesis–osteogenesis coupling, and bone formation/remodeling—regulated by PDGF/TGF-β/VEGF, BMP/Smad–VEGF–Wnt/β-catenin, and RANKL/OPG signaling, respectively. **(B)** Diabetes, smoking, infection, irradiation, and antiresorptive exposure disrupt distinct stages *via* persistent inflammation/ROS/impaired angiogenesis, clot instability/vasoconstriction/hypoxia, biofilm/chronic inflammation/bone destruction, hypovascularity–hypocellularity–hypoxia, and remodeling suppression/MRONJ risk. **(C)** Material functions map to specific windows: hemostasis/adhesion act early; antibiofilm/immunomodulation span clearance–transition; antioxidant support applies only to high-ROS pathological sites; structural support spans early-to-mid repair with shifting degradation/resorption; pro-angiogenic support acts from late inflammation through coupling; osteogenic support with controllable resorption applies to mid-to-late formation/remodeling.

### 3.3 Dominant regenerative barriers in pathological and medication-related high-risk conditions

Clinical pathological conditions do not merely slow the speed of socket healing; they can simultaneously alter the inflammatory response, redox balance, angiogenesis, cellular function, and the relationship between bone formation and resorption. DM, smoking, infection, radiotherapy, and medication exposure share some common downstream mechanisms, but their dominant barriers, severity, and intervention priorities differ (Gibon et al., 2017; Shen et al., 2021; Chavez et al., 2023; Popa et al., 2024; Yin et al., 2025).

#### 3.3.1 DM: impaired metabolic–immune–vascular coupling

The socket in diabetes is characterized by hyperglycemia-associated chronic inflammation, AGE accumulation, oxidative stress, microvascular dysfunction, and reduced MSC osteogenic capacity, which together impair inflammatory resolution, neovascularization, and bone matrix deposition (Oikawa et al., 2010; Silva et al., 2012; Wong et al., 2015; Caliaperoumal et al., 2018; Jiang et al., 2020; Doherty et al., 2021; Ko et al., 2021; Shen et al., 2021; Yin et al., 2021; Hofbauer et al., 2022; Meda Venkata et al., 2023; Pasarin et al., 2023; Li et al., 2024; Liu et al., 2024b; Xiong et al., 2025). Patients with diabetes are not homogeneous: glycemic control (e.g., HbA1c), disease duration, microvascular complications, medication, and local infection all influence outcomes. Diabetes-related material studies should therefore report metabolic-control indicators and interpret efficacy together with local infection and vascular status, rather than classifying patients simply as “diabetic” or “non-diabetic.”

#### 3.3.2 Smoking: vasoconstriction, tissue hypoxia, and oxidative damage

Smoking induces vasoconstriction, tissue hypoxia, oxidative damage, and osteoblast inhibition through nicotine and smoke particulates, while increasing clot instability and dry-socket risk (Ramp et al., 1991; Yuhara et al., 1999; Black et al., 2001; Feitelson et al., 2003; Barua et al., 2010; Giorgetti et al., 2012; Patel et al., 2013; Shah et al., 2019; Kuśnierek et al., 2022). Clinical impact depends on daily consumption, smoking duration, perioperative cessation, and coexisting periodontal infection; local materials may partly alleviate oxidative or vascular insufficiency but cannot offset sustained systemic smoking. Smoking exposure should therefore be reported as an independent variable rather than merged into a single pathological group.

#### 3.3.3 Infected sites: residual biofilm, persistent inflammation, and structural destruction

Periodontitis-, apical-periodontitis-, and abscess-associated sites present residual pathogens and mature biofilm, sustained inflammatory mediators, pre-existing bone-wall destruction, and impaired local cell function, often with complex wall defects and soft-tissue inflammation (Vijayan et al., 2014; Kim et al., 2016; Lee et al., 2016; Ben Amara et al., 2021; dos Santos et al., 2022; Dang et al., 2023; Alyousef and Almohaimeed, 2024; Lin et al., 2024; Mazurek-Mochol et al., 2024; Dai et al., 2025). Thorough debridement and infection-source control remain prerequisites for regeneration; antibacterial or anti-inflammatory materials are adjuncts, not substitutes for standard management (Kim et al., 2016; Ben Amara et al., 2021; Luan et al., 2023). Material design must address biofilm control, inflammation resolution, and structural reconstruction together, and should not be judged solely by short-term inhibition of a single planktonic strain.

#### 3.3.4 Post-irradiation sites: hypovascularity, hypocellularity, and long-term remodeling impairment

The irradiated jaw exhibits vascular injury, reduced cell number and viability, hypoxia, fibrosis, and weakened long-term remodeling; extraction further increases ORNJ risk, which depends on radiation dose, field, extraction timing, soft-tissue condition, and comorbidities (Marx et al., 1985; Marx and Johnson, 1987; Langley et al., 1997; Gal et al., 2000; Reuther et al., 2003; Szymczyk et al., 2004; de Arruda et al., 2006; Singh et al., 2012; Chandra et al., 2014; Owosho et al., 2017; Lajolo et al., 2021; Normando et al., 2022). Although experimental pro-angiogenic or osteocyte-protective strategies may aid repair (Karar and Maity, 2011; Donneys et al., 2016; Borges et al., 2023; Song et al., 2023), clinical management rests on risk stratification, minimally traumatic extraction, infection prevention, and long-term monitoring; experimental materials cannot replace standard ORNJ prevention.

#### 3.3.5 Medication-related high-risk sites: suppressed bone remodeling and the risk of MRONJ

Exposure to antiresorptive agents (bisphosphonates, denosumab) and certain antiangiogenic or immunomodulatory drugs inhibits osteoclast function and alters jaw remodeling and angiogenesis; AAOMS lists such therapy—alone or combined—as key background for MRONJ risk assessment (Ruggiero et al., 2022). Risk depends not on drug name alone but on indication, dose and route, cumulative exposure, underlying disease, concomitant glucocorticoid or chemotherapy, oral infection, and extraction trauma; oncology patients receiving high-dose antiresorptives generally carry higher risk than osteoporosis patients (Ruggiero et al., 2022; Schwech et al., 2023). Management should therefore prioritize medication/disease review, infection control, interdisciplinary consultation, minimally traumatic extraction, wound care, and long-term follow-up rather than empirical selection of strongly osteogenic or pro-angiogenic materials. No biomaterial currently has sufficient human evidence to prevent MRONJ independently; materials remain adjuncts to standard clinical management, and this review proposes no specific material prescription for this population.

#### 3.3.6 Shared mechanisms and condition-specific barriers

These conditions converge on persistent inflammation, oxidative stress, vascular insufficiency, and reduced osteogenesis, but their dominant barriers differ: diabetes on metabolic–immune–vascular coupling; smoking on vasoconstriction, hypoxia, and oxidation; infection on biofilm, persistent inflammation, and bone destruction; irradiation on reduced tissue viability and remodeling; and medication exposure on remodeling suppression and MRONJ risk (Gibon et al., 2017; Shen et al., 2021; Ruggiero et al., 2022; Chavez et al., 2023; Luan et al., 2023; Schwech et al., 2023; Li et al., 2024; Popa et al., 2024; Yin et al., 2025). Identifying the primary and secondary barriers at a given site is therefore more meaningful than selecting a uniform material by disease name; systemic risks that materials cannot correct require clinical management first, not compensatory functional stacking (condition-specific barriers and management boundaries are summarized in the pathological rows of Table 1).

**TABLE 1:** Multi-axis clinical assessment and material decision matrix for precision regeneration of the extraction socket.

Assessment dimension	Key variables/clinical phenotypes	Potential dominant regenerative barriers	Minimal necessary material functions	Clinical management and material boundaries	Recommended core outcomes	Representative references
Anatomical dimension	Intact four walls, continuous buccal bone plate, and favorable soft tissue conditions	The blood clot is affected by the open oral environment and local micromotion, but the regenerative space is generally stable	Blood clot protection, moderate osteoconduction, and early soft tissue sealing	Multifactor delivery or highly complex scaffolds should not be assumed necessary by default; whether to intervene depends on the subsequent restorative goals	Horizontal/vertical ridge changes, soft tissue contour, and pain and material exposure	(Hansson and Halldin, 2012; Tan et al., 2012; Jung et al., 2018; Majzoub et al., 2019; Stumbras et al., 2019; de Sousa Gomes et al., 2019; Guo et al., 2022; Udeabor et al., 2023; Marian et al., 2024)
Anatomical dimension	Thin buccal bone plate or insufficient continuity	Programmed resorption of the external contour and reduced stability of the blood clot/graft particles	Volume stability, particle fixation, and moderate barrier and soft tissue support	Bone plate thickness can only serve as a risk indicator; fixed thresholds should not be applied in isolation from tooth position, soft tissue, and surgical trauma	Buccal contour, horizontal/vertical dimensions, and the proportion of new bone to residual material	(Matarasso et al., 2009; Avila-Ortiz et al., 2014; Cardaropoli et al., 2014; Spinato et al., 2014; de Carvalho Formiga et al., 2019; Kim et al., 2021; Steigmann et al., 2022; ElNahass et al., 2024; Fok et al., 2024; Ekhlasmandkermani et al., 2025; Ghanaati et al., 2025; Madi et al., 2025)
Anatomical dimension	Three-wall, two-wall, or more complex bone wall defects	Collapse of the regenerative space, soft tissue invasion, material migration, and restricted vascular access routes	Barrier membrane, form maintenance, scaffold/membrane fixation, interconnected pores, and vascularization support	Only complex defects are likely to benefit from individualized scaffolds; regular four-wall sites generally do not require individualized fabrication	Defect fill, contour stability, need for secondary bone augmentation, and suitability for implant placement	(Avila-Ortiz et al., 2014; Goh et al., 2015; Juodzbalys et al., 2019; Bartnikowski et al., 2020; Kim et al., 2021; Anderson et al., 2022; Steigmann et al., 2022; Elbanna et al., 2023; Ivanovski et al., 2024; Ekhlasmandkermani et al., 2025)
Pathological dimension	DM or poor metabolic control	Hyperglycemia/AGE-related inflammation, NETosis, oxidative stress, microangiopathy, MSC senescence, and impaired osteogenesis (local barriers: excessive ROS, persistent inflammation, vascular insufficiency, reduced cellular function)	First ensure structural stability; then select antioxidant, immunomodulatory, pro-angiogenic, or osteogenic support according to the specific barriers	Materials cannot replace glycemic and metabolic control, infection management, or complication assessment. Direct evidence from materials in human extraction sockets is limited; most evidence is mechanistic, derived from general bone defects, or based on animal studies. Studies should report HbA1c/metabolic status, infection, and microvascular complications	Vascular perfusion, inflammation/ROS, new bone maturity, and infection and complications	(Oikawa et al., 2010; Silva et al., 2012; Wong et al., 2015; Filion et al., 2017; Caliaperoumal et al., 2018; Jiang et al., 2020; Doherty et al., 2021; Ko et al., 2021; Shen et al., 2021; Yin et al., 2021; Hofbauer et al., 2022; Meda Venkata et al., 2023; Pasarin et al., 2023; Li et al., 2024; Liu et al., 2024b; Xiong et al., 2025)
Pathological dimension	Smoking and sustained tobacco exposure	Nicotine/smoke-induced vasoconstriction, hypoxia, abnormal clot architecture, excessive ROS, oxidative damage, and osteogenic inhibition (local barriers: blood clot instability, excessive ROS, insufficient local perfusion)	Blood clot reinforcement; antioxidant or pro-angiogenic adjuncts when necessary	Local materials cannot offset sustained smoking; dosage, years of smoking, and perioperative smoking cessation should be reported. Direct animal socket evidence exists, but the clinical compensatory effect of materials remains insufficient	Dry socket/infection, blood clot stability, vascularization, and trabecular reconstruction	(Ramp et al., 1991; Yuhara et al., 1999; Black et al., 2001; Feitelson et al., 2003; Barua et al., 2010; Giorgetti et al., 2012; Patel et al., 2013; Shah et al., 2019; Vo et al., 2020; Kuśnierek et al., 2022; Wang et al., 2024; Esfahrood et al., 2025; Zhao et al., 2025)
Pathological dimension	Infected sites associated with periodontitis, apical periodontitis, or abscess	Multispecies biofilm, sustained inflammatory mediators, pre-existing bone wall destruction, and impaired local cellular function (local barriers: recolonization by residual biofilm, persistent inflammation, structural defects)	Post-debridement antibiofilm activity + timely inflammation resolution + space maintenance	Antibacterial materials cannot replace mechanical removal of infected and necrotic tissue and infection source control. Direct evidence from infected extraction sockets should be prioritized; in vitro antibacterial effects against a single planktonic strain can only serve as mechanistic evidence	Mature biofilm, clinical infection, inflammatory cell composition, and new bone and soft tissue repair	(Vijayan et al., 2014; Kim et al., 2016; Lee et al., 2016; Ben Amara et al., 2021; dos Santos et al., 2022; Dang et al., 2023; Luan et al., 2023; Alyousef and Almohaimeed, 2024; Lin et al., 2024; Mazurek-Mochol et al., 2024; Dai et al., 2025)
Pathological dimension	Sites after head and neck radiotherapy	Microvascular injury, reduced cellularity, hypoxia, fibrosis, and impaired long-term remodeling (local barriers: soft tissue fragility, insufficient perfusion, impaired cell survival and bone maturation)	Soft tissue protection under minimally traumatic conditions; potential pro-angiogenic/cytoprotective support as adjuncts	ORNJ risk stratification, infection prevention, minimally traumatic extraction, and long-term monitoring should be performed first. Pro-angiogenic or osteogenic strategies are supported mainly by experimental evidence and cannot be extrapolated as standard treatment	Wound dehiscence, infection/necrosis, vascularization, bone maturation, and long-term safety	(Marx et al., 1985; Marx and Johnson, 1987; Langley et al., 1997; Gal et al., 2000; Reuther et al., 2003; Szymczyk et al., 2004; de Arruda et al., 2006; Karar and Maity, 2011; Singh et al., 2012; Chandra et al., 2014; Donneys et al., 2016; Owosho et al., 2017; Lajolo et al., 2021; Normando et al., 2022; Borges et al., 2023; Song et al., 2023)
Pathological dimension	Exposure to antiresorptive, antiangiogenic, or related medications	Antiresorptive agents suppress osteoclasts and bone remodeling; some medications affect vascular and soft tissue repair (local barriers: insufficient wound healing, risks of infection and osteonecrosis)	In principle, materials serve only as adjuncts to standard management; no specific experimental material prescription is proposed	Verify the indication, dose, route, cumulative exposure, infection, and concomitant medications; multidisciplinary assessment. Insufficient human evidence that any material can independently prevent MRONJ.	MRONJ, wound healing, infection, and long-term follow-up	(Biguetti et al., 2019; Ruggiero et al., 2022; Schwech et al., 2023)
Surgical dimension	Flap elevation/flapless procedures, extraction trauma, debridement, material compaction, and fixation	Altered periosteal blood supply, intraoperative bone wall injury, pore compression, and material displacement	Fixation and coverage matched to the intraoperative defect; avoidance of excessive compaction	Material dosage, packing method, membrane type, fixation, and closure mode should be reported	Soft tissue thickness, pain, material stability, bone wall status, and complications	(Barone et al., 2013; Barone et al., 2015; Lim et al., 2019; Couso-Queiruga et al., 2021; Kim et al., 2021; Steigmann et al., 2022; Siu et al., 2023; Song et al., 2024b; Ekhlasmandkermani et al., 2025)
Restorative dimension	Esthetic zone, planned implant timing, and whether subsequent bone augmentation is acceptable	Different clinical objectives lead to different requirements for contour and mature bone volume	Selection of the lowest necessary complexity according to the restorative goals	The value of a material should be judged by whether it improves the ideal implant position and reduces secondary surgery and patient burden	3D implant position, primary stability, secondary bone augmentation, and patient-reported outcomes and costs	(Hansson and Halldin, 2012; Tan et al., 2012; Jung et al., 2018; Avila-Ortiz et al., 2019; Stumbras et al., 2019; de Carvalho Formiga et al., 2019; Canullo et al., 2022; Guo et al., 2022)

In this table, “minimal necessary material functions” refers to the functions that must be prioritized before proceeding to material design; it does not imply that all functions should be stacked at all sites.

### 3.4 Modulatory effects of surgical and restorative contexts on material efficacy

*In vivo* material performance is shaped not only by composition but by extraction approach, soft-tissue management, infection clearance, material placement, and the restorative plan. Intraoperative wall injury can convert an intact socket into a structural defect, and excessive curettage, compaction, or manipulation may impair local blood supply; extraction trauma and intraoperative wall status should therefore be fundamental variables when interpreting material efficacy (Kim et al., 2021; Steigmann et al., 2022; Song Y. et al., 2024; Ekhlasmandkermani et al., 2025).

Flap elevation facilitates exposure, debridement, and barrier fixation but may compromise periosteal blood supply; flapless procedures reduce tissue stripping but demand better exposure assessment and fixation. Human studies show no consistent short-term hard-tissue difference between flap and flapless ARP, whereas soft-tissue thickness, pain, and keratinized tissue may differ (Barone et al., 2015; Siu et al., 2023).

Closure mode likewise affects material retention and soft-tissue repair: primary closure reduces exposure and particle loss but may alter vestibular depth and keratinized-mucosa position, whereas open healing reduces traction but depends on covering-material stability and infection control (Lim et al., 2019). No closure mode is absolutely superior independent of material and site conditions.

Material placement also modulates efficacy: underpacking fails to maintain contour, overpacking reduces pore interconnectivity and vascular entry, and inadequate membrane fixation leads to displacement. Studies should report dosage, packing method, membrane type, fixation, closure mode, and maintenance to allow valid comparison (Barone et al., 2013; Barone et al., 2015; Lim et al., 2019; Couso-Queiruga et al., 2021; Siu et al., 2023).

The restorative plan defines the clinical objective: esthetic-zone and implant-planned sites emphasize buccal contour, soft-tissue thickness, and 3D implant position, whereas sites not planned for implants may not require complex materials. Efficacy should be judged against planned implant timing, prosthetic approach, and reduced secondary augmentation rather than short-term radiographic bone volume alone.

### 3.5 From multi-axis clinical phenotyping to biomaterial functional requirements

Material selection should not be reduced to a single anatomical indicator or disease label; four dimensions—anatomical, pathological, surgical, and restorative—should be evaluated simultaneously (Hansson and Halldin, 2012; Tan et al., 2012; Avila-Ortiz et al., 2014; Barone et al., 2015; Gibon et al., 2017; Jung et al., 2018; Avila-Ortiz et al., 2019; Lim et al., 2019; Stumbras et al., 2019; Kim et al., 2021; Ko et al., 2021; Shen et al., 2021; Canullo et al., 2022; Guo et al., 2022; Kuśnierek et al., 2022; Ruggiero et al., 2022; Steigmann et al., 2022; Chavez et al., 2023; Luan et al., 2023; Siu et al., 2023; Popa et al., 2024; Song Y. et al., 2024; Ekhlasmandkermani et al., 2025; Yin et al., 2025). The specific variables of each dimension are shown in Figure 2B.

These factors may coexist in the same patient—for example, a patient with DM may present an infected socket with a thin buccal plate. Material decision-making should therefore distinguish primary barriers, secondary barriers, and risk factors that materials cannot correct: the primary barrier determines the core functions a material must possess, secondary barriers determine whether additional functions are warranted, and uncontrolled systemic disease, sustained smoking, residual infection, or high-risk medication exposure cannot be compensated for by material functions in place of clinical management.

Integrating the anatomical, pathological, surgical, and restorative factors, the regenerative barriers of extraction sockets can be summarized into five categories: instability of the blood clot and regenerative space, persistent infection or inflammation, oxidative stress overload, insufficient angiogenesis, and osteogenesis–bone remodeling imbalance (Gibon et al., 2017; Shen et al., 2021; Chavez et al., 2023; Luan et al., 2023; Li et al., 2024; Popa et al., 2024; Yin et al., 2025). These barriers may coexist, but their severity and priority of intervention differ.

Insufficient clot or structural stability is the fundamental barrier at wall-defect and thin-plate sites, which first require hemostasis, adhesion, and space maintenance. Infected or persistently inflamed sites require biofilm control on the basis of thorough debridement plus timely inflammation resolution. DM- and smoking-related sites may benefit from limiting excessive oxidative stress without abolishing the redox signaling required for repair. Post-irradiation, severely metabolic, and extensive-defect sites should prioritize vascular and cell survival conditions rather than simply increasing osteogenic factors.

Material design for extraction sockets thus follows the hierarchy of “structural stability first, infection and inflammation under control, vascularization prioritized, and osteogenesis matched to degradation,” operationalized as: assess the four dimensions → identify primary and secondary barriers → exclude non-correctable risks → determine the minimal necessary function combination → select the lowest-effective-complexity material → define mechanistic, tissue-level, and clinical endpoints. This process aligns with Figure 2 and Table 1; Section 4 examines how material systems realize these functions and the trade-offs of functional stacking.

## 4 Mechanism-oriented biomaterial design and functional implementation for the extraction socket

Section 3 demonstrates that bone regeneration in the extraction socket is influenced by multiple factors, including the stability of the blood clot and regenerative space, infection and inflammation, oxidative stress, angiogenesis, and the balance between osteogenesis and bone remodeling. No material is absolutely superior or inferior independent of the clinical scenario; its value depends on whether it can provide the necessary functions at the appropriate time against the dominant barriers of a specific site. Therefore, this section no longer takes natural polymers, synthetic polymers, bioceramics, and composite materials as the primary classification basis, but instead analyzes materials around the functions of blood clot stabilization, space maintenance, microbial and immune modulation, osteogenesis–angiogenesis coupling, pathological microenvironment responsiveness, and anatomical adaptation. Within this framework, natural and synthetic materials are regarded as engineering modules for achieving specific functions rather than as independent therapeutic categories.

### 4.1 Blood clot stabilization, rapid hemostasis, and wet-environment adhesion

Early materials must provide rapid hemostasis and clot stabilization while promoting a continuous, stable soft-tissue barrier over the wound: prolonged exposure of the socket or material increases particle loss and infection risk, whereas excessive flap traction for primary closure may displace the keratinized mucosa. Materials must therefore balance clot protection, epithelial migration, and connective-tissue formation against the ingrowth of deep vasculature and bone-forming tissue (Barone et al., 2013; Barone et al., 2015; Lim et al., 2019; Couso-Queiruga et al., 2021; Siu et al., 2023).

Suitable materials should combine rapid fluid absorption or *in situ* gelation, moderate wet adhesion, washout resistance, and soft-tissue compatibility, without impeding deep-tissue and vascular ingrowth through excessive swelling, sustained acidification, or an overly dense barrier (Kong et al., 2004; Ma et al., 2020; Toledano et al., 2020; Zhang et al., 2020; Xie et al., 2021; Li S. et al., 2023; Qian et al., 2024; Agnes et al., 2025; Kanagal et al., 2025; Pintilei et al., 2025). Evaluation should extend beyond clotting time and fluid absorption to wound epithelialization, soft-tissue thickness, material exposure, pain, infection, and subsequent bone formation.

Collagen and its derivatives are widely used in extraction sockets and guided bone regeneration (GBR): collagen mimics part of the extracellular matrix (ECM), supports platelet adhesion, fibrin-network stabilization, and cell migration, and can be fabricated into sponges, membranes, or hydrogels (Linsenmayer et al., 1978; Zitzmann et al., 1997; Toledano et al., 2020; Xie et al., 2021; Agnes et al., 2025). Non-reinforced collagen, however, has limited mechanical stability and may degrade too rapidly in protease-rich inflamed environments, whereas cross-linking can reduce cell-adhesion-site accessibility or increase cytotoxicity. The design focus should be the balance among clot protection, soft-tissue repair, cell infiltration, and degradation rate rather than degradation prolongation alone.

Chitosan is cationic and interacts with cell membranes, mucins, and negatively charged microbial surfaces, conferring hemostatic, adhesive, and antibacterial potential (Fakhri et al., 2020; Confederat et al., 2021; Li et al., 2022; Flores-Espinoza et al., 2023; Elshahat et al., 2024; Miranda et al., 2025). Its functions vary with molecular weight, deacetylation degree, substituents, and pH, and most studies target planktonic or non-oral strains; its efficacy against multispecies socket biofilms still requires direct verification.

Oxidized cellulose promotes hemostasis through fluid absorption, packing, and local acidification (Zhang et al., 2020; Li S. et al., 2023; Kanagal et al., 2025), but sustained low pH may impair osteoblast function and calcium-phosphate stability, so its value lies in short-term hemostasis rather than long-term osteoconduction. Alginate forms ionically cross-linked hydrogels with tunable mechanics and degradation (Kong et al., 2004; Ma et al., 2020; Qian et al., 2024; Pintilei et al., 2025) but lacks cell-adhesion sites and usually requires collagen, gelatin, RGD sequences, or mineral components to improve tissue integration.

#### 4.1.1 Autologous blood-derived matrices: blood clot reinforcement and early soft tissue repair

Autologous blood-derived materials include platelet-rich plasma (PRP), platelet-rich fibrin (PRF), and their variants. PRF-type materials form a dense fibrin network carrying platelets, leukocytes, and repair-related mediators; their autologous origin, short preparation time, and clot/membrane form suit coronal coverage, clot reinforcement, and early soft-tissue repair (Pan et al., 2019; Canellas et al., 2020; Castro et al., 2021; Encalada Abad et al., 2023; Siawasch et al., 2025).

Human studies support PRF for improving early postoperative pain, wound healing, and certain soft- and hard-tissue indicators, but its effects on long-term ridge width, height, and new-bone proportion are inconsistent (Pan et al., 2019; Canellas et al., 2020; Castro et al., 2021; Encalada Abad et al., 2023; Siawasch et al., 2025); some randomized studies found L-PRF improved healing indicators, whereas others found 3D ridge preservation close to that of the natural clot. PRF should therefore not be indiscriminately described as replacing bone substitutes with long-term space-maintenance capability.

PRF composition varies with collection tube, centrifugation speed and time, rotor radius, and processing interval, so L-PRF, A-PRF, A-PRF+, and CGF are not identical materials; studies should fully report preparation parameters and distinguish PRF used alone, as a covering membrane, or combined with bone substitutes.

Functionally, PRF’s clearest advantages lie in clot reinforcement, early soft-tissue healing, and patient experience; its long-term space maintenance is limited. It is a reasonable low-complexity option for intact four-wall sockets, but at thin-plate or wall-defect sites it should serve only as an adjunct to bone substitutes or barrier systems.

Evaluation of early hemostatic and soft-tissue materials should include wet-environment retention, stability after salivary flushing, clot preservation, epithelialization, soft-tissue thickness, pain, exposure, and subsequent bone formation—not merely clotting time. For routine intact sockets, a simple material that stabilizes the clot and promotes soft-tissue sealing may already suffice; complex multifactor delivery systems need not be introduced prematurely.

### 4.2 Regenerative space maintenance, osteoconduction, and degradation adaptation

When a socket presents a thin buccal plate or wall defects, the material’s primary task shifts from clot protection to maintaining regenerative space and ridge contour: it must hold volume and pore interconnectivity under soft-tissue contraction while allowing gradual vascular, cellular, and bony ingrowth. Premature degradation collapses the space; excessively slow degradation leaves residual particles, foreign-body reactions, or delayed maturation (Dumas et al., 2014; Avila-Ortiz et al., 2019; Canullo et al., 2022; Tajvar et al., 2023).

#### 4.2.1 Remodeling characteristics and applicability boundaries of commonly used clinical bone substitute materials

Clinical ARP fillers include autologous, allogeneic, xenogeneic, and synthetic bone substitutes. Their value cannot be judged by osteoconductivity alone; volume stability, new-bone formation, residual particles, degradation, source-related risks, operability, and implant requirements must be weighed together (Araújo et al., 2015; Avila-Ortiz et al., 2019; Majzoub et al., 2019; Canullo et al., 2022; Chisci et al., 2023).

Autologous bone carries endogenous cells and biological activity but requires a donor site and may remodel rapidly, so it is not the default for routine socket preservation. Allogeneic bone avoids a second site but varies with donor and processing. Xenogeneic bone offers good volume stability but higher long-term particle retention. Synthetic substitutes are compositionally controllable, but their dissolution and replacement still depend on crystalline phase, porosity, and sintering.

Meta-analytic evidence quantifies the efficacy of ARP in limiting dimensional loss: compared with unassisted healing, socket grafting preserves a mean of 1.89 mm (95% CI: 1.41–2.36 mm; *p* < 0.001) in buccolingual width and 2.07 mm (95% CI: 1.03–3.12 mm; *p* < 0.001) in midbuccal height (Avila-Ortiz et al., 2014). Histomorphometric meta-analyses further demonstrate that the histological advantage of ARP is biomaterial-dependent; while overall ARP does not guarantee a higher proportion of vital bone than spontaneous healing (mean difference −5.86%, 95% CI: −13.84%–2.11%; *p* = 0.15), autologous platelet concentrates can increase vital bone by a mean of 16.28% (p < 0.0001), and the choice of graft material and membrane application significantly modulates residual graft content (Benekou et al., 2025). Xenografts and allografts frequently leave considerable residual particles (15%–36% at approximately 5–6 months), whereas synthetic biomaterials tend to substitute more rapidly (Schnutenhaus et al., 2018; Canullo et al., 2022; Ozzo and Kheirallah, 2024). Therefore, it is inappropriate to determine a “best material” applicable to all sites based on a single indicator; the optimal choice depends on whether the clinical priority is maximal ridge dimension preservation (mm) or fastest complete bone replacement (% vital bone *versus* % residual graft). Sites with thin buccal bone plates or in the esthetic zone may place greater emphasis on volume stability; sites planned for earlier implant placement may place greater emphasis on the material replacement rate; and sites with poor soft tissue conditions also need to consider safety after material exposure.

Clinical comparisons should simultaneously report ridge dimensions, newly formed bone, residual material, and the proportion of connective tissue. If a material maintains the contour mainly through long-term residual particles, “ridge contour maintenance” and “complete bone replacement” should be clearly distinguished, and radiographically dense areas should not be directly interpreted as mature functional new bone.

#### 4.2.2 Barrier membranes and wound coverage strategies

Barrier membranes restrict premature entry of epithelial and non-osteogenic tissue and help fix particulate grafts. Resorbable collagen membranes avoid secondary removal but their stability and degradation depend on source, cross-linking, and the inflammatory environment (Zitzmann et al., 1997; Ghanaati, 2012; Toledano et al., 2020; Abtahi et al., 2023); non-resorbable d-PTFE membranes provide a more stable barrier but require removal and careful management of exposure and infection (de Carvalho Formiga et al., 2019; Couso-Queiruga et al., 2021).

Membrane selection should match defect morphology and closure mode: intact four-wall sockets may need only a collagen plug or fibrin coverage, whereas thin-plate or wall-defect sites rely more on membrane–particle composites; under open healing the covering must limit particle loss, and under primary closure flap tension and keratinized-mucosa changes must be considered (Barone et al., 2013; Lim et al., 2019; Couso-Queiruga et al., 2021).

Membranes are not passive covers: their pores, thickness, degradation, and surface properties affect protein adsorption, inflammation, and vascular entry, and too-rapid or too-slow degradation each carries risk. Membrane and filler should be evaluated as an interdependent space-maintenance system.

#### 4.2.3 Structure–degradation balance of synthetic polymers and calcium phosphate materials

PLGA and PCL can be tuned through molecular weight, polymer ratio, pore structure, and manufacturing (Shive and Anderson, 1997; Gunatillake and Adhikari, 2003; Ostafinska et al., 2015). PLGA offers processability and drug loading but its acidic degradation products may lower local pH; PCL is mechanically stable and slow-degrading, favoring long-term space maintenance but potentially delaying tissue replacement. Clinical evidence for PLGA scaffolds in sockets remains markedly thinner than for conventional bone substitutes (Hoda et al., 2016).

HA and β-TCP are the common calcium phosphate bioceramics: HA is osteoconductive and volume-stable but slow to degrade, whereas β-TCP dissolves and is replaced faster, with actual behavior depending on crystalline phase, porosity, sintering, and cellular activity (LeGeros, 2002; Lu and Zreiqat, 2010; Jeong et al., 2019; Tanvir et al., 2024; Sadlik et al., 2025; Saxena et al., 2025).

Direct socket studies show calcium phosphates and their composites can support ridge preservation and new bone, but compositions, defects, and evaluation times vary widely (Ho et al., 2016; Ozzo and Kheirallah, 2024; Kim D. W. et al., 2025); human replacement behavior cannot be inferred from the theoretical Ca/P ratio or *in vitro* dissolution alone.

#### 4.2.4 Performance complementarity and complexity costs of composite materials

Polymer–ceramic composites combine polymer formability and toughness with a calcium-phosphate osteoconductive interface. Direct socket animal studies have evaluated systems such as HA/β-TCP–collagen, and general bone-engineering studies show hybrid systems can overcome single-material limitations (Yeo and Kim, 2012; Nie et al., 2015; Dhand et al., 2016; Ho et al., 2016; Farjaminejad et al., 2024; Hamdy et al., 2025; Kim D. W. et al., 2025).

Composite components, however, complicate degradation kinetics, batch consistency, and sterilization stability; studies should report new bone, residual material, and connective tissue rather than judging efficacy by micro-CT bone volume or ridge dimensions alone.

Space maintenance is thus the combined result of modulus, pore interconnectivity, degradation, particle fixation, barrier membranes, closure, and soft-tissue pressure. Direct human evidence remains concentrated on conventional bone substitutes, barrier membranes, and a few polymers; most novel composite scaffolds are supported mainly by animal or general bone-defect studies.

### 4.3 Antibiofilm activity, inflammation control, and immunomodulation

Material design for infection-related sites must address both microbial burden and host inflammation. Oral infections persist as multispecies biofilms whose bacteria tolerate antimicrobials, immune clearance, and environmental change, so judging materials by inhibition zones or short-term bactericidal rates against single planktonic strains overestimates their real anti-infective capability (Halkai et al., 2017; Fakhri et al., 2020; Li et al., 2020; Confederat et al., 2021; Li et al., 2022; Flores-Espinoza et al., 2023; Liang et al., 2023; Elshahat et al., 2024; Holguín-Meráz et al., 2024; Miranda et al., 2025).

Chitosan, antimicrobial peptides, and Ag, Cu, or Zn components can confer antibacterial activity (Gabriel et al., 2006; Choe et al., 2015; Halkai et al., 2017; Fakhri et al., 2020; Confederat et al., 2021; Li et al., 2022; Soma et al., 2022; Flores-Espinoza et al., 2023; Liang et al., 2023; Elshahat et al., 2024; Holguín-Meráz et al., 2024; Miranda et al., 2025; Monje et al., 2025), but a narrow window may separate effective antibacterial concentrations from those safe for host cells: higher doses raise bactericidal rates yet may inhibit MSCs, osteoblasts, and endothelial cells. Antibacterial materials should be evaluated simultaneously against multispecies biofilms, host cells, and angiogenesis, not by bactericidal rate alone.

Infection control cannot replace standard debridement either. For extraction sockets associated with apical periodontitis, periodontal abscess, or severe periodontitis, mechanical removal of necrotic tissue and infection sources remains the prerequisite for regeneration. Materials are mainly used to reduce recolonization by residual microorganisms, limit biofilm recovery, and improve the local microenvironment after debridement, and should not be described as being able to eliminate infection alone.

The goal of inflammation modulation is not complete suppression: early neutrophil and macrophage responses are required for pathogen clearance and repair initiation, and only persistent or uncontrolled inflammation inhibits vascularization and osteogenesis. IL-4, anti-inflammatory small molecules, NF-κB-related modulators, and immunoactive materials can shift macrophage phenotype and cytokine expression (Li Y. et al., 2023; Huang et al., 2024; Chen et al., 2025; Eom et al., 2025; Qi et al., 2025; Shi et al., 2025), but the M1-to-M2 transition cannot be simplified as “the faster the better.”

Therefore, anti-infective and immunomodulatory materials should follow the principle of “first controlling the infectious burden, then promoting timely inflammation resolution.” Their key evaluation indicators should include the mature biofilm burden, local inflammatory cell composition, dynamic changes in macrophages, vascular formation, osteoblast function, and the final bone tissue quality.

### 4.4 Osteogenesis–angiogenesis coupling and bioactive factor delivery

Bone formation in the socket depends on spatiotemporal coupling of angiogenesis, cell recruitment, and osteogenic differentiation. For intact, well-perfused sockets, calcium phosphates and stable matrices may suffice; for post-irradiation, DM, or extensive wall-defect sites, insufficient angiogenesis may become limiting before osteogenic signals do. Factor delivery should therefore respect the vascularization–osteoinduction–degradation sequence rather than simply pursuing stronger osteogenesis.

Recombinant human bone morphogenetic protein-2 (rhBMP-2) is among the few growth factors evaluated in socket clinical studies: combined with an absorbable collagen carrier it may improve bone formation at certain socket or buccal-defect sites (Fiorellini et al., 2005; Coomes et al., 2014). Its efficacy is strongly dose- and carrier-dependent, and higher doses or rapid release can cause local inflammation, edema, aberrant bone, and added cost (Fiorellini et al., 2005; Coomes et al., 2014; Raina et al., 2016; Geng et al., 2021; Onji et al., 2021; Chernysheva et al., 2025; Surisaeng et al., 2025), so higher BMP-2 loading is not simply better design.

VEGF promotes endothelial migration, proliferation, and lumen formation, providing the vascular basis for osteoblast survival. Combined or sequential BMP-2/VEGF delivery has shown synergy in general bone-defect and jaw models (Young et al., 2009; Wang et al., 2018; Liu et al., 2020; Tong et al., 2023; Khoswanto and Dewi, 2025), but doses, carriers, and models differ too much to derive an optimal ratio for sockets, and simultaneous burst release may misalign the two signals’ effective windows.

Carriers decisively shape growth-factor efficacy and safety: collagen, calcium phosphates, hydrogels, and polymer microspheres modulate release through adsorption, ionic interaction, encapsulation, or covalent binding (Fiorellini et al., 2005; Young et al., 2009; Coomes et al., 2014; Zhang et al., 2015; Raina et al., 2016; Wang et al., 2018; Liu et al., 2020; Geng et al., 2021; Onji et al., 2021; Tong et al., 2023; Chernysheva et al., 2025; Khoswanto and Dewi, 2025; Surisaeng et al., 2025). An ideal carrier limits early burst release, protects activity, and sustains effective dosing after vascular and osteoblastic entry; over-strong binding restricts release, whereas over-rapid degradation risks local high-dose exposure.

Evaluation of osteogenesis–angiogenesis coupling materials should jointly report vascular density or perfusion, osteoblast differentiation, new-bone maturity, and factor safety; for routine sites without vascular impairment, combined BMP-2/VEGF delivery should not be assumed superior to simple osteoconductive materials.

### 4.5 Healing sequence adaptation and pathological microenvironment responsiveness

Healing-sequence-adapted materials match functions to events such as clot stabilization, inflammatory transition, angiogenesis–osteogenesis coupling, and remodeling: hierarchical structures, core–shell particles, and multi-carrier combinations can release components in a preset sequence (Dashtimoghadam et al., 2020; Ma et al., 2020; Zhang et al., 2025; Zheng et al., 2025; Qiu et al., 2026). Such release is predetermined by structure and formulation, however—“programmed” or “sequential” release should not be equated with closed-loop systems that sense and adjust in real time.

Pathology-responsive materials use acidic pH, excess ROS, or MMP activity as triggers, altering degradation and release through hydrazone, boronic-ester, disulfide, or enzymatically cleavable bonds (Chen et al., 2021; Lin et al., 2022; Wei et al., 2022; Chen et al., 2023; Liu X. et al., 2025; Liu F. et al., 2025). *In vitro* and general bone-defect studies show such designs can modulate anti-inflammatory, antioxidant, antibacterial, or osteogenic release under preset stimulation, but stable “on-demand treatment” in human sockets remains unproven.

The core limitation is the temporal, spatial, and inter-individual variability of pathological signals: pH, ROS, and enzyme activity differ across DM, smoking, infection, and irradiation, and gradients exist within a single socket. Thresholds set too high fail to trigger; too low, release fires during normal early inflammation. Post-cleavage degradation products, post-sterilization responsiveness, storage stability, and batch consistency also require verification (Lin et al., 2022; Wei et al., 2022; Chen et al., 2023; Liu F. et al., 2025).

Animal socket studies have begun evaluating novel hydrogels (Guo et al., 2026), but most pH-, ROS-, and MMP-responsiveness evidence comes from *in vitro*, skin-wound, or general bone-defect models. Clinical “intelligence” cannot be proven at a single pH or fixed H_2_O_2_ concentration; triggers, release, and long-term safety must be verified in models incorporating saliva, biofilm, proteases, oxidative stress, and mechanical disturbance.

### 4.6 Anatomical adaptation, surface topology, and 3D printing

Structural design spans three levels: macroscopic morphology (stable defect filling and contour maintenance), the pore network (cell migration, nutrient exchange, vascular ingrowth), and micro-/nanoscale surface topology (integrin-, focal-adhesion-, and mechanotransduction-mediated regulation of adhesion and osteogenesis) (Juodzbalys et al., 2019; Bertrand et al., 2020; Zhu et al., 2020; Jiao et al., 2023; Ma et al., 2024).

Micro-/nanoscale roughness, microgrooves, and nanofibers affect cell alignment, cytoskeletal tension, and osteogenic differentiation, possibly *via* integrin–PI3K/Akt, FAK/RhoA/YAP, and PIEZO1 mechanotransduction (Chang et al., 2018; Bertrand et al., 2020; Zheng et al., 2020; Zhu et al., 2020; Yang J. et al., 2025); these effects are material-, cell-, and scale-dependent, and parameters from titanium surfaces cannot be directly extrapolated to degradable socket scaffolds.

3D printing can fabricate CBCT-matched scaffolds with controlled pores, mechanics, and component distribution (Bartnikowski et al., 2020; Freeman et al., 2021; Anderson et al., 2022; Slavin et al., 2024), and pilot ARP and complex-defect studies suggest translational potential (Goh et al., 2015; Ivanovski et al., 2024); however, human evidence remains small-sample or case-based and cannot prove general superiority over particulate grafts and membranes.

Pore design has no single “optimal size”: larger interconnected pores favor vascular and tissue ingrowth but weaken structure, whereas smaller pores favor capillary action but may limit cell entry. General scaffold studies support interconnectivity and gradient structures (Lee et al., 2019; Jiao et al., 2023; Li L. et al., 2023; Yang et al., 2023), but specific parameters require verification in alveolar and socket models.

Therefore, 3D printing is more suitable for three-wall or two-wall defects or complex alveolar bone defects in which conventional particulate materials can hardly maintain the space stably. For regularly shaped four-wall intact extraction sockets, the manufacturing time, cost, sterilization, and quality control burdens added by individualized printing may not necessarily generate additional clinical net benefit.

### 4.7 Boundaries of functional integration and evidence evaluation

Multifunctional materials can provide hemostatic, antibacterial, antioxidant, immunomodulatory, pro-angiogenic, and osteogenic effects simultaneously, but more functions do not necessarily yield additive efficacy: different functions may have conflicting dose and time windows. The key to complex material design is avoiding mutual interference, not maximizing function count.

Antibacterial ions, nanoparticles, or active molecules must reach effective local concentrations that may also damage MSCs, osteoblasts, and endothelial cells; sustained or excessive ROS scavenging may interfere with immune defense and signaling; premature suppression of pro-inflammatory responses may impede clearance of infection and necrotic tissue; and overly strong or rapid pro-angiogenic signals may increase abnormal vascular permeability.

Multi-factor or multi-drug systems may face carrier-binding competition, misaligned release kinetics, component inactivation, and alteration of the local environment by degradation products; sequential release is superior to simpler materials only when the effective windows of each function have been directly verified.

Trade-offs also exist in material structure. Increasing the cross-linking degree and mechanical strength favors space maintenance, but may reduce pore interconnectivity, cell infiltration, and degradation rate; increasing macropores favors vascular entry but may weaken structural stability; and prolonging material retention favors contour maintenance but may delay complete replacement by new bone.

Multifunctional materials should therefore follow the “lowest necessary complexity” principle: for each added function, the targeted clinical barrier, effective dose, action window, component compatibility, and added tissue or clinical benefit should be clarified. Function modules and their engineering trade-offs are shown in Figure 4, and site applicability, evidence directness, and translational limitations of each material category are summarized in Supplementary Table S2.

**FIGURE 4:**
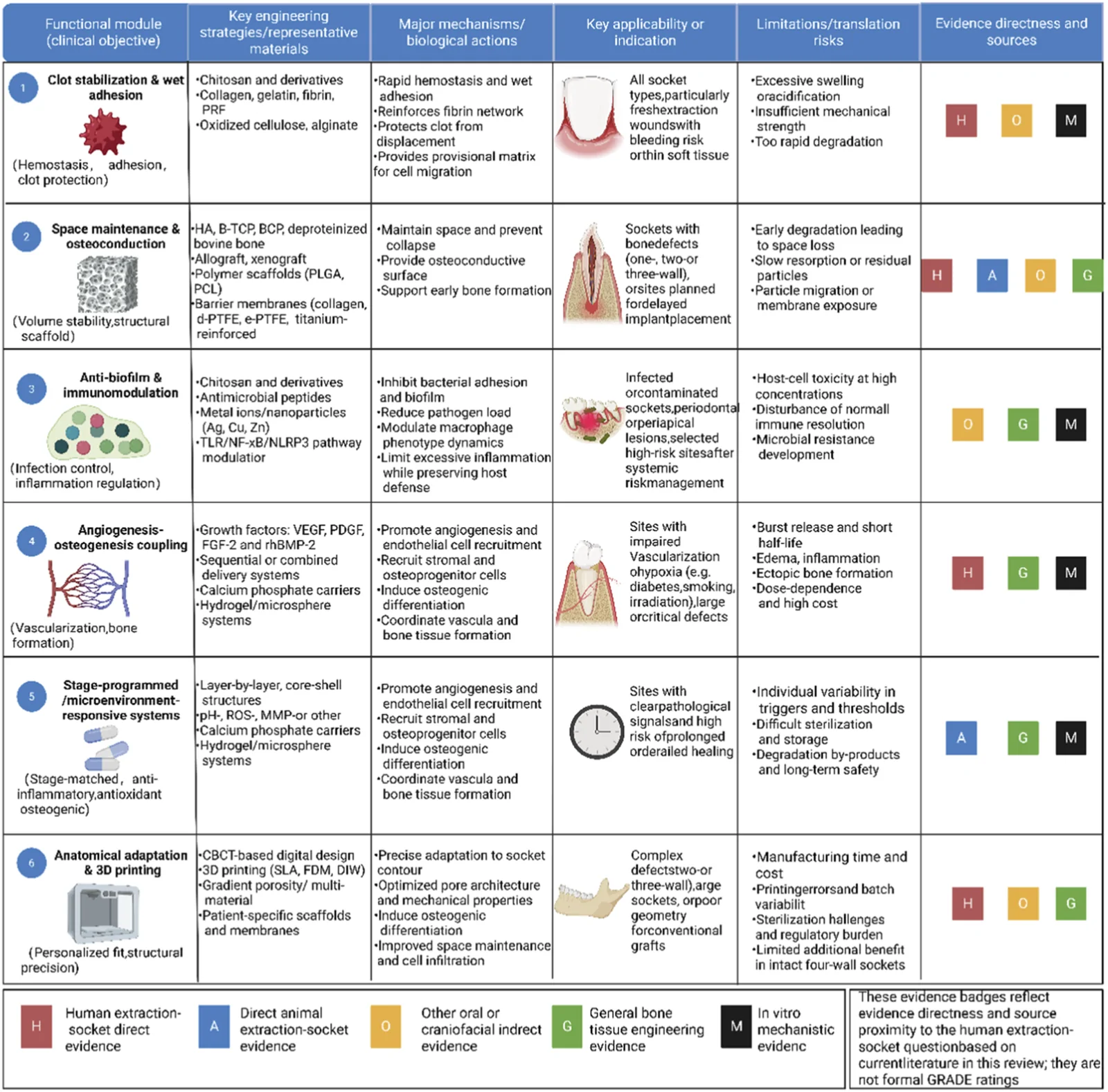
Function-oriented biomaterial toolbox, engineering trade-offs, and evidence directness for precision socket regeneration. Six functional modules are summarized: (1) blood-clot stabilization and wet adhesion—chitosan, collagen, gelatin, fibrin, PRF, oxidized cellulose, and alginate provide rapid hemostasis and a provisional matrix but risk swelling, weak mechanics, and fast degradation; (2) space maintenance and osteoconduction—HA, β-TCP/BCP, allo-/xenogeneic bone, PLGA, PCL, and membranes preserve space and guide ingrowth but risk premature loss, particle persistence or migration, or membrane exposure; (3) antibiofilm and immunomodulation—chitosan, antimicrobial peptides, metallic ions (Ag, Cu, Zn), and TLR/NF-κB/NLRP3-targeted strategies reduce pathogen burden and modulate inflammation, requiring a balance between antibacterial efficacy and host-cell safety; (4) angiogenesis–osteogenesis coupling—VEGF, PDGF, FGF-2, rhBMP-2, and calcium-phosphate/hydrogel/microsphere carriers promote vascular and osteogenic ingrowth but carry burst-release, edema, ectopic bone, dose, and cost risks; (5) programmed/pathology-responsive systems—layer-by-layer, core–shell, dynamic-covalent, and pH-/ROS-/MMP-responsive designs enable staged or triggered release but face uncertain human trigger thresholds, sterilization/storage difficulties, and long-term safety gaps; (6) anatomical adaptation and 3D printing—CBCT-based patient-specific scaffolds/membranes improve adaptation in complex defects but add time, cost, error, and regulatory burden, with little added benefit for routine intact sockets. H, A, O, G, and M denote direct human-socket, animal-socket, other intraoral/craniomaxillofacial, general bone-engineering, and *in vitro* mechanistic evidence, respectively; this is not a formal GRADE rating. Material selection should follow the “lowest necessary complexity” matched to dominant site barriers.

## 5 From material design to clinical decision-making: evidence maturity, translational boundaries, and future research priorities

The preceding sections moved from socket phenotype, pathological microenvironment, and healing sequence to material functions including clot stabilization, space maintenance, antibiofilm activity, immunomodulation, osteogenesis–angiogenesis coupling, and pathological responsiveness. Possessing a function, however, does not make a material suitable for every socket: clinical translation requires judging whether the function targets the site’s dominant barrier and whether the added benefit offsets the safety, cost, and operational burdens of complexity. This section therefore establishes phenotype-based selection principles, distinguishes evidence maturity, and identifies priority questions for future research.

### 5.1 Clinical material selection based on extraction socket phenotype

#### 5.1.1 Healthy routine extraction sockets with intact bone walls

Healthy sockets with intact walls have stable clot boundaries, sufficient osteogenic-cell sources, and vascular access; material intervention mainly aims to reduce ridge volume loss, stabilize the clot, and preserve soft- and hard-tissue contours for subsequent restoration, rather than to correct a pathological microenvironment (Hansson and Halldin, 2012; Tan et al., 2012; Jung et al., 2018; Majzoub et al., 2019; Stumbras et al., 2019; Guo et al., 2022; Marian et al., 2024).

For such sites, collagen sponges, collagen membranes, and conventional osteoconductive bone substitutes already have a robust clinical basis (Linsenmayer et al., 1978; Zitzmann et al., 1997; Ghanaati, 2012; Ho et al., 2016; Majzoub et al., 2019; Chisci et al., 2023; Ozzo and Kheirallah, 2024; Kim D. W. et al., 2025). Meta-analytic data show that even simple grafting reduces the need for secondary augmentation—implants can be placed at the planned 3D position without further augmentation in ∼90.1% of ARP-treated *versus* 79.2% of unassisted sockets—and the mean dimensional benefit across conventional materials is approximately 1.31–1.54 mm horizontally and 0.91–1.12 mm vertically (Willenbacher et al., 2016), generally sufficient for routine intact sockets. Selection should therefore prioritize operational simplicity, stability, predictable degradation, and no added complications; antioxidant, gene-delivery, or multi-factor sequential-release functions should not be assumed necessary by default, because excessive functional design increases cost and quality-control difficulty and may introduce cytotoxicity and uncontrolled-release risks.

Whether ARP is indicated at all should also follow the restorative plan: if the site does not affect the planned 3D implant position, esthetic contour, or denture support, the net benefit of intervention may be limited. Material selection should serve defined restorative goals rather than treating every socket as a defect requiring complex regeneration.

#### 5.1.2 Sites with thin buccal bone plates and bone wall defects

Sockets with thin buccal plates, discontinuous walls, or frank wall defects are prone to clot destabilization, material migration, soft-tissue invasion, and contour collapse (Avila-Ortiz et al., 2014; Cardaropoli et al., 2014; Spinato et al., 2014; de Carvalho Formiga et al., 2019; Kim et al., 2021; Steigmann et al., 2022; ElNahass et al., 2024; Ekhlasmandkermani et al., 2025; Madi et al., 2025); the primary requirement is maintaining space and external contour, not boosting early osteogenic markers.

Thin-plate sites need moderate volume stability without tissue compression from excessive stiffness, overpacking, or swelling; three-wall, two-wall, or complex defects may require particulate substitutes combined with barrier membranes, fixation structures, or form-stable scaffolds to resist soft-tissue contraction (Goh et al., 2015; Wang et al., 2015; Lee et al., 2019; Bartnikowski et al., 2020; Dong et al., 2021; Freeman et al., 2021; Anderson et al., 2022; Liu et al., 2022; Song et al., 2022; Dalfino et al., 2023; Gao et al., 2023; Yang et al., 2023; Ivanovski et al., 2024; Liu et al., 2025d; Müller et al., 2025).

CBCT can preoperatively assess wall integrity, plate thickness, and defect extent and aid selection of material volume and morphology (Song Y. et al., 2024; Ekhlasmandkermani et al., 2025), but imaging cannot replace judgment of infection, soft-tissue blood supply, systemic status, and intraoperative wall injury. Individualized 3D printing has value in complex defects, yet for regular four-wall sockets its time, cost, and regulatory burden may not yield additional benefit (Huang et al., 2013; Yuan et al., 2017; Garot et al., 2020; Freeman et al., 2021; Ahmed et al., 2025).

#### 5.1.3 Boundaries of material selection for pathological and medication-related high-risk sites

The common feature of sites associated with infection, DM, smoking, radiotherapy, and antiresorptive medication exposure is that material efficacy is highly dependent on whether the underlying risks are controlled. Material selection at such sites should be built on thorough debridement, metabolic management, smoking cessation intervention, radiotherapy or medication risk assessment, and necessary interdisciplinary collaboration. Materials can improve local regenerative conditions, but cannot replace the management of pathogenic factors.

For infected sites, antibiofilm and moderate immunomodulatory functions may be added after thorough debridement; for DM- and smoking-related sites, antioxidant or pro-angiogenic functions according to oxidative and vascular impairment; and for post-irradiation sites, minimally traumatic procedures, soft-tissue protection, and risk monitoring come first. Patients exposed to antiresorptive or antiangiogenic drugs should be stratified by indication, dose, course, route, and infection status, and no specific regenerative material prescription should be proposed without direct human evidence (Ruggiero et al., 2022; Schwech et al., 2023).

Material studies at such sites should report glycemic control, smoking exposure, radiation dose and field, medication type and route, infection status, and surgical complexity; without these variables, material effects cannot be separated from the underlying disease or procedures.

#### 5.1.4 Multi-axis clinical assessment and the principle of the lowest necessary complexity

Material selection should integrate all four dimensions: the anatomical dimension determines space and structural-support requirements; the pathological dimension determines the need for anti-infective, immunomodulatory, antioxidant, or pro-angiogenic functions; the surgical dimension affects blood supply, material stability, and closure; and the restorative dimension defines the clinical objectives. These assessments feed the precision-regeneration framework of Section 3.5, which matches the minimal necessary function combination to site-specific barriers at the lowest effective complexity.

Sites with intact four walls and no obvious pathological barriers mainly require blood clot stabilization and moderate osteoconduction; sites with thin buccal bone plates and bone wall defects prioritize space maintenance; infected sites should add antibiofilm functions on the basis of thorough debridement; and only DM, smoking, or post-irradiation sites may further integrate antioxidant, immunomodulatory, or pro-angiogenic functions according to the dominant barriers.

Material functions should be added stepwise according to clinical necessity, rather than using the number of functions as the evaluation criterion for advancement. For uncontrolled infection, metabolic abnormalities, sustained smoking, radiotherapy-related high risk, or the risk of MRONJ, the underlying risks must be addressed first, rather than compensating by increasing material complexity.

### 5.2 Evidence maturity and extrapolation boundaries of biomaterials

#### 5.2.1 Material systems with an established clinical application basis

Collagen membranes and sponges, xenogeneic and allogeneic bone, synthetic calcium phosphates, and their composites are the most widely used ARP materials (Linsenmayer et al., 1978; Zitzmann et al., 1997; Ghanaati, 2012; Ho et al., 2016; Avila-Ortiz et al., 2019; Majzoub et al., 2019; Canullo et al., 2022; Chisci et al., 2023; Ozzo and Kheirallah, 2024; Kim D. W. et al., 2025); their main clinical value is reducing ridge dimensional change, maintaining implantation space, and providing stable conditions for subsequent implant placement.

However, dimensional preservation of the alveolar ridge should not be directly equated with an increase in functional new bone. Slowly degrading materials may maintain volume through residual particles, while histologically the proportions of new bone, residual material, and connective tissue may present different results. Therefore, radiographic contour preservation and histological bone replacement should be regarded as complementary but non-equivalent endpoints.

rhBMP-2 with a collagen carrier is among the few bioactive systems evaluated in human sockets or related alveolar defects (Boyne et al., 2005; Fiorellini et al., 2005; Triplett et al., 2009; Coomes et al., 2014; Jung et al., 2022): it may promote bone formation in certain complex defects, but efficacy varies with dose, carrier, and defect type and is accompanied by inflammation, swelling, aberrant bone, and cost issues, so it cannot be generalized as routine socket standard care.

#### 5.2.2 Technologies at the preclinical or early translational stage

Pathology-responsive hydrogels, gene-engineered cells, and viral vectors remain at the *in vitro* or animal stage, with manufacturability, sterilization stability, and clinical net benefit unclear (Gafni et al., 2004; Park et al., 2018; Freitas et al., 2019; Kim et al., 2019; Park et al., 2019; Freitas et al., 2021; Wei et al., 2022). 3D-printed scaffolds have higher translational maturity, though evidence remains limited to small samples (Goh et al., 2015; Bartnikowski et al., 2020; Freeman et al., 2021; Ivanovski et al., 2024).

Emerging technologies—AI-assisted CBCT segmentation, CRISPR-engineered MSCs, AAV-mediated gene delivery, and organ-on-a-chip systems—have exploratory value but face substantial translational barriers: AI optimizes segmentation accuracy rather than outcome prediction (Domic et al., 2021; Abesi et al., 2023; Omigbodun and Oladapo, 2025; Yang F. et al., 2025); CRISPR-MSCs and AAV vectors carry immune, regulatory, and safety challenges without direct human socket evidence (Gafni et al., 2004; Park et al., 2018; Freitas et al., 2019; Kim et al., 2019; Park et al., 2019; Freitas et al., 2021); and organ-on-a-chip models remain simplified *in vitro* environments that do not replicate the open socket (Khashayar et al., 2017; França et al., 2020; Yang et al., 2021; Jayawardena et al., 2026). These are best positioned as long-term research tools, not near-term clinical alternatives.

#### 5.2.3 Distinguishing evidence sources, study designs, and clinical translational levels

Evidence in this field should not be summarized by a single “high–low level” hierarchy; instead, the directness of the evidence source with respect to the clinical problem of human extraction sockets, the internal validity of the study design, and the distance between the study endpoint and clinical net benefit should be evaluated separately.

By evidence source and extrapolation distance, studies fall into five categories (denoted H, A, O, G, and M): direct human socket evidence (RCTs, prospective and histological studies, and their systematic reviews); direct animal socket evidence (rodent, canine, minipig socket models); indirect evidence from other intraoral or jawbone models (periodontal defects, peri-implant augmentation, sinus elevation); general bone tissue engineering evidence (calvarial, long-bone, subcutaneous models); and *in vitro* mechanistic evidence (cell, biofilm, release-kinetics, degradation, and responsiveness experiments).

Evidence-source directness is not equivalent to study quality: case reports are direct human evidence but may carry higher bias risk than well-designed animal studies, and RCT, cohort, or review labels describe design, not source. Efficacy judgments should also weigh sample size, controls, randomization and blinding, outcome completeness, follow-up, and heterogeneity. Endpoints should be evaluated separately as well: cell viability, osteogenic genes, ROS, cytokines, and short-term micro-CT bone fraction are mechanistic surrogates; ridge dimensions, new bone, residual material, connective tissue, and soft-tissue contour are tissue-level outcomes; and secondary-augmentation need, planned implant position, primary stability, complications, patient-reported outcomes, time, and cost approach clinical net benefit. Improvement at a lower level cannot be automatically inferred as higher-level benefit.

Evidence from different sources and endpoints cannot be used interchangeably: osteogenic, pro-angiogenic, antibacterial, or anti-inflammatory effects seen in general bone-defect or *in vitro* studies support only a potential mechanism, not effectiveness in human sockets exposed to saliva, biofilm, and mechanical disturbance. Accordingly, claims in this review are phrased by evidence source (“studies in human extraction sockets have shown,” “animal socket studies suggest,” and so on). The translational pathway and its extrapolation boundaries are shown in Figure 5, and the evidence maturity of different materials is summarized in Table 2.

**FIGURE 5:**
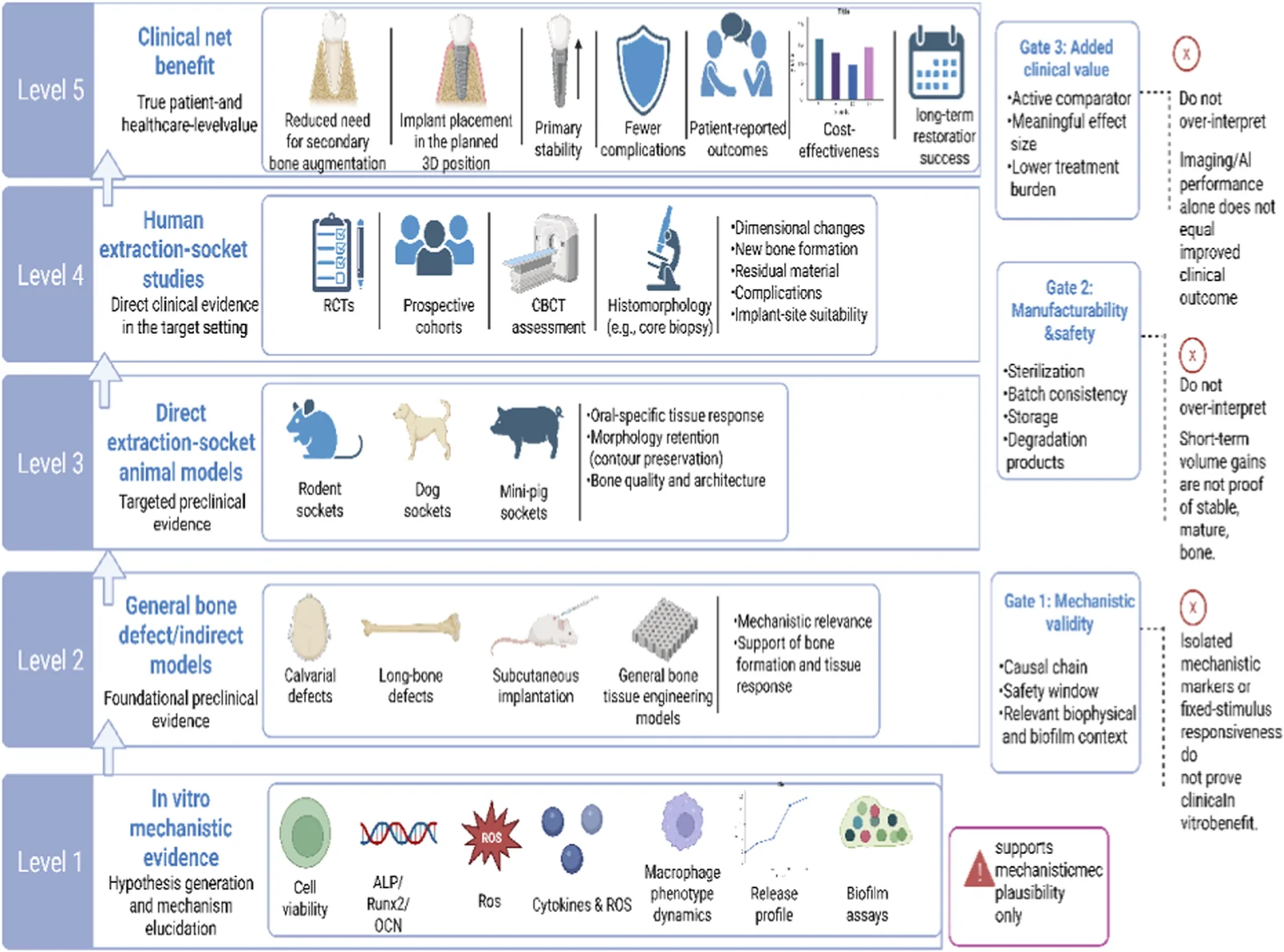
Five-level translational pathway from mechanistic research to clinical net benefit, with key translational gates; levels are not a single evidence-quality hierarchy. Level 1, *in vitro* mechanistic evidence (cell viability, ALP/Runx2/OCN, ROS/cytokines, macrophage phenotype, release profiles, biofilm assays), generates hypotheses but cannot prove clinical efficacy. Level 2, general bone-defect/indirect models (calvarial/long-bone defects, subcutaneous implantation), assess mechanism and safety windows but lack the open, saliva-/biofilm-/loading-exposed socket environment. Level 3, direct animal socket models (rodent, canine, minipig), evaluate oral-specific responses, morphological adaptation, contour preservation, and bone quality. Level 4, direct human socket research (RCTs, prospective cohorts, CBCT, histomorphometry), assesses ridge dimensions, new bone, residual material, complications, and implant-site suitability. Level 5, clinical net benefit (reduced secondary augmentation, planned 3D implant position, primary stability, fewer complications, patient-reported outcomes, cost-effectiveness, long-term prosthetic success).

**TABLE 2:** Evidence maturity, outcome hierarchy, and clinical translational positioning of biomaterial technologies for the extraction socket.

Evidence tier	Typical data/indicators	Technology/material examples	Can support	Cannot support	Key next steps	Representative references
Preclinical: *in vitro* mechanistic and indirect *in vivo* evidence	Cell viability; ALP, Runx2, OCN; ROS/inflammatory cytokines; macrophage phenotype; release profiles; biofilm assays; calvarial/long-bone defect and subcutaneous outcomes	Smart responsive, antibacterial, immunomodulatory, and pro-angiogenic/osteogenic delivery systems; multifactor delivery, mechanotransduction, gene-engineered MSCs/AAV	Hypothesis generation, material screening, mechanisms of action, basic tissue responses, and safety windows	Performance in the open, saliva-/biofilm-/loading-exposed socket; human ARP efficacy or patient benefit	Combine release, stimulus-intensity, and cytotoxicity data; establish human pathological-signal thresholds; progress to oral socket models	(Gafni et al., 2004; Chang et al., 2018; Park et al., 2018; Elkassih et al., 2019; Freitas et al., 2019; Kim et al., 2019; Park et al., 2019; Bertrand et al., 2020; Dashtimoghadam et al., 2020; Zheng et al., 2020; Zhu et al., 2020; Chen et al., 2021; Freitas et al., 2021; Lin et al., 2022; Wei et al., 2022; Chen et al., 2023; Li et al., 2023d; Liu et al., 2025c; Oh et al., 2025; Yang et al., 2025b; Zheng et al., 2025; Qiu et al., 2026)
Direct animal socket evidence	Ridge contour, bone volume/quality, material remnants, and soft tissue in rodent, canine, or minipig sockets	HA/β-TCP composites, composite hydrogels, and antioxidant/immunomodulatory/pro-angiogenic systems	Oral-specific tissue responses, morphological adaptation, and mechanistic feasibility	Human ARP efficacy, reduced secondary augmentation, or patient benefit	Large-animal oral models with biofilm and mechanical loading; long-term material remnants	(Giorgetti et al., 2012; Ho et al., 2016; Kim et al., 2016; Shen et al., 2021; Borges et al., 2023; Dang et al., 2023; Ma et al., 2024; Hamdy et al., 2025; Khoswanto and Dewi, 2025; Kim et al., 2025b; Guo et al., 2026)
Established clinical application basis	Ridge dimensions, tissue composition, material remnants, and implantation conditions	Collagen membranes/sponges, xenogeneic bone, allogeneic bone, synthetic calcium phosphate materials, and combinations	Ridge/contour outcomes and implantation conditions in human sockets	Radiographic contour preservation cannot be equated with complete bone replacement or long-term implant benefit	Active controls, unified histological indicators, and long-term implant and patient outcomes	(Linsenmayer et al., 1978; Zitzmann et al., 1997; Boyne et al., 2005; Triplett et al., 2009; Ghanaati, 2012; Avila-Ortiz et al., 2014; Avila-Ortiz et al., 2019; Majzoub et al., 2019; Avila-Ortiz et al., 2020; Couso-Queiruga et al., 2021; Canullo et al., 2022; Jung et al., 2022; Chisci et al., 2023; Ozzo and Kheirallah, 2024; Madi et al., 2025)
Clinical adjunct with heterogeneous effects	Blood-clot retention, early soft-tissue healing, and patient experience	PRF/autologous blood-derived matrices	Clot reinforcement, early soft-tissue healing, and partial improvement of patient experience	Stable long-term ridge-volume maintenance or replacement of bone grafts	Standardized centrifugation parameters; distinguish single-use/coverage/combined application; long-term 3D outcomes	(Pan et al., 2019; Canellas et al., 2020; Castro et al., 2021; Encalada Abad et al., 2023; Siawasch et al., 2025)
Limited direct human evidence	Bone formation or space maintenance at selected complex sites	rhBMP-2/collagen carrier, PLGA scaffolds, and 3D-printed PCL or patient-specific scaffolds	Potential benefit at selected complex sites	Routine standard treatment for all sockets; superiority over established membrane–particle systems	Enlarged samples, active standard-treatment controls, dose/carrier safety, and cost-effectiveness	(Boyne et al., 2005; Fiorellini et al., 2005; Triplett et al., 2009; Coomes et al., 2014; Goh et al., 2015; Hoda et al., 2016; Jung et al., 2022; Ivanovski et al., 2024)
Research tools and long-term translational platforms	Segmentation accuracy and screening/monitoring throughput	AI/CBCT, microfluidics, bone/tooth-on-a-chip, smart sensing, and closed-loop designs	Efficiency of segmentation, material screening, mechanistic monitoring, and design	Replacement of clinical judgment; segmentation accuracy alone does not prove improved outcomes	Multicenter external validation, real clinical endpoints, open oral-microenvironment models, and regulatory pathways	(Khashayar et al., 2017; França et al., 2020; Domic et al., 2021; Yang et al., 2021; Huang et al., 2022; Abesi et al., 2023; Cinici et al., 2024; Kumar et al., 2025; Omigbodun and Oladapo, 2025; Raji et al., 2025; Vo and Trinh, 2025; Yang et al., 2025a; Zuo et al., 2025; Gkantidis et al., 2026; Jayawardena et al., 2026; Liu et al., 2026)
Clinical and patient-relevant outcomes	Need for secondary augmentation, planned 3D implant position, primary stability, implant/prosthetic outcomes, infection, dehiscence, exposure, pain, satisfaction, treatment time, and cost	All materials entering human studies, especially multifunctional and individualized systems	True clinical net benefit of the material	Mechanistic gains not translated into reduced treatment burden cannot justify material complexity	Active controls with collagen membranes or common bone substitutes; report effect sizes and complications	(Boyne et al., 2005; Fiorellini et al., 2005; Triplett et al., 2009; Hansson and Halldin, 2012; Tan et al., 2012; Coomes et al., 2014; Jung et al., 2018; Avila-Ortiz et al., 2019; Stumbras et al., 2019; de Carvalho Formiga et al., 2019; Canullo et al., 2022; Guo et al., 2022; Jung et al., 2022; Kumar et al., 2024)

Function-specific minimum requirements—space-maintenance materials should report contour + residual material + secondary augmentation; antibacterial materials should use mature-biofilm, clinical-infection, and host-cell outcomes; pro-angiogenic materials should report perfusion + mature bone; responsive materials should demonstrate a stimulus–response–release–tissue-benefit causal chain.

Clinical translation requires passing three sequential gates: mechanistic effectiveness (causal chain, safety window, relevance to the oral biophysical and biofilm environment); manufacturability and safety (sterilization, batch consistency, storage stability, degradation products); and clinical added value (active controls, clinically meaningful effect sizes, reduced treatment burden). *In vitro* osteogenic gains, short-term bone-volume increases, improved imaging or AI metrics, and responsive release under fixed stimulation cannot be directly extrapolated to clinical benefit. Complex materials are justified only when mechanistic advantages translate into stable, reproducible, clinically meaningful net benefit.

### 5.3 Necessary conditions for clinical evaluation and translation

#### 5.3.1 Establishing a clinically relevant multidimensional outcome system

Outcomes should be classified by distance from ultimate clinical benefit into mechanistic surrogate indicators, tissue and anatomical indicators, and clinical and patient-relevant outcomes; improvement at a lower level cannot automatically imply higher-level benefit (Boyne et al., 2005; Fiorellini et al., 2005; Triplett et al., 2009; Hansson and Halldin, 2012; Tan et al., 2012; Barone et al., 2013; Coomes et al., 2014; Jung et al., 2018; Avila-Ortiz et al., 2019; Stumbras et al., 2019; de Carvalho Formiga et al., 2019; Canullo et al., 2022; Guo et al., 2022; Jung et al., 2022; Fok et al., 2024; Gabay et al., 2024).

Endpoints should match intended function: space-maintenance materials should focus on contour, remnants, and secondary augmentation; antibacterial materials on mature biofilm and clinical infection rather than inhibition zones; pro-angiogenic materials on perfusion plus mature bone; and responsive materials on demonstrating the signal–response–release–tissue-benefit causal chain.

Future clinical studies should use active controls—collagen membranes, common bone substitutes, or their combinations—rather than only proving superiority to natural healing. Complexity has clear clinical significance only when mechanistic or histological gains translate into fewer secondary surgeries, better implant position, fewer complications, or improved patient experience. Minimum evaluation requirements and recommended active controls per function are summarized in Table 2.

#### 5.3.2 Manufacturability, sterilization, and quality control

Laboratory performance translates clinically only if composition, pores, mechanics, degradation, and release remain consistent after scale-up; each added component—cells, growth factors, nanoparticles, gene carriers—raises batch-variation and quality-control demands (Nuss and von Rechenberg, 2008; Vo et al., 2012; Shim et al., 2013; Dumas et al., 2014; Filippi et al., 2020; Wei et al., 2020; Bazgir et al., 2021; Hunckler and Levine, 2022; Namanloo et al., 2022; Shi et al., 2022; Utami Nike et al., 2022; Abtahi et al., 2023; Jee et al., 2023; Tajvar et al., 2023; Zhou et al., 2023; Liu et al., 2024a; Verykokou et al., 2024; Wahba, 2024; Zhao et al., 2024; Liu et al., 2025b; Shao et al., 2025; Su et al., 2025; Tian X. et al., 2025; Wu et al., 2025; Xiang et al., 2025; Tousidonis et al., 2026).

Sterilization may alter polymer molecular weight, collagen structure, growth factor activity, and the stability of responsive bonds. Material research should specify the sterilization method and compare mechanics, degradation, release, and biological activity before and after sterilization. Injectable or *in situ* gel-forming materials also need to be evaluated for viscosity, cross-linking ability, and sterile packaging stability over the storage period.

For 3D-printed or individualized scaffolds, quality control cannot rely solely on design files; printing errors, pore interconnectivity, actual mechanical properties, residual monomers, and implantation fit should also be verified (Zhou et al., 2023; Verykokou et al., 2024; Zhao et al., 2024; Shao et al., 2025; Tian X. et al., 2025; Tousidonis et al., 2026).

#### 5.3.3 Long-term safety and regulatory boundaries

The long-term safety of materials depends on the source of raw materials, degradation products, residual particles, immune responses, and the local microbial environment. The absence of obvious short-term inflammation in animal studies cannot replace the evaluation of long-term foreign body reactions, bone maturation, and systemic toxicity (Spalazzi et al., 2008; Thrivikraman et al., 2014; Vasconcelos et al., 2015; Linn et al., 2023; Maduka et al., 2023; Nonhoff et al., 2024; Wan et al., 2024; Aikin et al., 2025; Carballo-Pedrares et al., 2026).

Materials containing growth factors, cells, viral vectors, or drugs may be classified as combination products, whose regulatory requirements are usually higher than those of simple bone substitute materials. When a function is added to a material, it should be clarified whether that function produces a measurable clinical gain and whether the newly introduced risks can be managed through standardized testing and manufacturing control.

#### 5.3.4 Clinical operability and cost-effectiveness

Clinical materials need to accommodate limited operation time and saliva- and blood-contaminated environments, and allow clinicians to achieve stable implantation across different defect morphologies. Excessive reliance on dedicated equipment, complex mixing steps, or long setting times reduces the feasibility of materials in routine clinical practice (Huang et al., 2013; Yuan et al., 2017; Garot et al., 2020; Song Y. et al., 2024; Zhao et al., 2024; Ahmed et al., 2025).

Modular materials may offer a compromise between individualization and scale-up. For example, standardized membranes, particles, and injectable components can be combined to achieve different functions, rather than remanufacturing a complete scaffold for each patient. Individualized printing should be prioritized for complex bone wall defects in which conventional materials can hardly maintain space.

Cost-effectiveness evaluation should not only compare the unit price of materials, but should also incorporate operation time, the need for secondary bone augmentation, management of complications, and the difficulty of subsequent implant restoration. Complex materials have reasonable clinical economics only when they can reduce additional treatment or significantly improve patient outcomes.

### 5.4 Research questions requiring priority resolution in the future

#### 5.4.1 Establishing real in vivo thresholds of pathological signals

For pH-, ROS-, and MMP-responsive materials, the core question is not whether they respond *in vitro* but whether signal intensity in human sockets stably reaches the designed threshold; future work should map pathological-signal ranges across patients, tooth positions, and healing time points (Elkassih et al., 2019; Dashtimoghadam et al., 2020; Lin et al., 2022; Wei et al., 2022; Chen et al., 2023; Li W. et al., 2023; Du et al., 2025; Liu F. et al., 2025; Oh et al., 2025; Shen et al., 2025; Liu et al., 2026; Qiu et al., 2026).

In the absence of real *in vivo* data, *in vitro* testing at a fixed pH or a high H_2_O_2_ concentration can only prove that a material is chemically responsive, and cannot prove that it can release on demand in clinical extraction sockets.

#### 5.4.2 From bone formation alone to coordinated bone–soft tissue regeneration

Clinical outcomes after extraction depend not only on the intra-socket bone volume, but are also influenced by the buccal contour, soft tissue thickness, keratinized mucosa, and wound closure. Future materials should consider the competition between rapid soft tissue migration and bone formation, and evaluate whether materials can promote a stable soft tissue barrier while maintaining the space for bone regeneration.

The optimal material structure, degradation rate, and growth factor requirements of bone and soft tissue are not entirely consistent. Hierarchical or gradient structures have theoretical advantages, but it needs to be demonstrated that they provide additional clinical benefit over simple membrane–bone substitute combinations, rather than merely improving *in vitro* markers.

#### 5.4.3 Establishing experimental models with greater predictive power

Rodent extraction sockets are suitable for early mechanistic research, but their healing speed, alveolar bone structure, and occlusal environment differ markedly from those of humans. Before entering clinical application, complex materials should be preferentially verified in large-animal models that can simulate alveolar bone anatomy, the oral microbiota, and the mechanical environment.

Microfluidics, organ-on-a-chip, and multicellular co-culture can be used to simulate the interactions among salivary flow, biofilm, inflammatory cells, vascular endothelium, and osteoblasts (Khashayar et al., 2017; França et al., 2020; Yang et al., 2021; Jayawardena et al., 2026). The value of these models lies mainly in preventing materials without translational potential from entering animal experiments, rather than completely replacing animal and human studies.

#### 5.4.4 Clarifying the auxiliary boundaries of AI in clinical decision-making

AI can support CBCT segmentation, wall measurement, defect classification, and scaffold modeling, but its clinical value depends on multicenter external validation, interpretability, and outcome prediction (Domic et al., 2021; Abesi et al., 2023; Cinici et al., 2024; Omigbodun and Oladapo, 2025; Yang F. et al., 2025; Gkantidis et al., 2026); studies should verify whether AI-assisted decisions reduce measurement error, improve material selection, or lower secondary-augmentation rates—not report segmentation accuracy alone.

AI is more suitable as a tool for phenotype identification and design assistance, rather than replacing clinicians’ comprehensive judgment of infection, soft tissue, systemic disease, and the restorative plan.

#### 5.4.5 Comparing the true net benefit of complex materials with standard treatment

Studies of novel materials usually use blank scaffolds or no-material groups as controls, but the question that the clinic truly needs to answer is whether complex materials are superior to currently common collagen membranes, bone substitute materials, or their combination.

Future research should adopt clinically meaningful active controls and report:

- clinical effect sizes10;
- complications;
- material remnants;
- the need for secondary bone augmentation;
- patient-reported outcomes;
- costs; and
- long-term implant restoration outcomes.

Only when multifunctional materials provide stable, reproducible, clinically meaningful net benefit over standard treatment is their complexity justified; these questions determine whether novel materials can progress from mechanistic feasibility to manufacturable, verifiable options with clinical added value.

### 5.5 Limitations of this review

This is a critical narrative review, not a systematic review or meta-analysis; literature selection, thematic synthesis, and the “site phenotype–dominant barrier–minimal necessary functions” framework inevitably reflect the authors’ judgment, so the decision pathway should be read as an evidence-based heuristic rather than a rigid clinical algorithm.

The included studies are highly heterogeneous in models (rodent, canine, minipig, human), materials, manufacturing parameters, surgical protocols, defect morphologies, evaluation time points, and endpoints, precluding quantitative pooling or formal GRADE rating; comparative statements are therefore qualitative and contextual, not hierarchical rankings.

Direct human socket evidence remains limited or absent for several emerging strategies—including responsive hydrogels, multifactor sequential delivery, gene-engineered cell therapies, and patient-specific 3D-printed scaffolds—so conclusions for these technologies rely on extrapolation from *in vitro* studies, general bone-defect models, or small pilot trials.

The proposed framework, though grounded in current evidence and interdisciplinary experience, has not been prospectively validated in large RCTs or multicenter cohorts; its clinical utility, inter-rater reliability, and cost-effectiveness require further study.

Finally, the boundaries between evidence-source categories are not absolute: calvarial or long-bone studies may still inform material–cell interactions, and some human ARP studies have small samples or incomplete histological outcomes. Readers should weigh evidence directness alongside study-design quality when interpreting our conclusions.

## 6 Conclusion

The extraction socket is not a simple bony cavity with uniform regenerative potential; its healing is jointly shaped by anatomical phenotype, pathological microenvironment, surgical procedures, soft-tissue conditions, restorative objectives, and healing sequence. No single optimal material exists for all sockets, and clinical value cannot be judged by function count or short-term osteogenic indicators.

Precision regeneration requires identifying dominant barriers across the anatomical, pathological, surgical, and restorative dimensions and delivering only the minimal necessary functions, temporally matched to healing. Clot and space stabilization remain the foundation; infection control and timely inflammation resolution are prerequisites at pathological sites; vascularization gates cell survival and sustained osteogenesis in compromised sites; and degradation must synchronize with new-bone formation. Risks that materials cannot correct—systemic disease, sustained smoking, residual infection, irradiation, medication-related risk—should be managed clinically first, not by adding material complexity.

Material evaluation should shift from mechanistic surrogates to clinically relevant endpoints—ridge dimensions, tissue composition, soft-tissue contour, implant-site suitability, secondary-augmentation need, complications, patient-reported outcomes, and cost. Future work should define the real spatiotemporal ranges of pathological signals in human sockets, adopt models closer to the open oral environment, and use standard materials as active controls; complex designs are translationally rational only when added functions yield stable, reproducible, clinically meaningful net benefit.
