# Supplementary material for: Biomaterial strategies for precision regeneration of the tooth extraction socket: integrating site phenotype, pathological microenvironment, and healing sequence
Source: Front Bioeng Biotechnol. 2026 Sep 21;14:1957296. doi: 10.3389/fbioe.2026.1957296 (PMC13635737; doi:10.3389/fbioe.2026.1957296)
Supplement: Supplementary file 1 [file DataSheet1.pdf]

Supplementary Materials

Biomaterial Strategies for Precision Regeneration of the Tooth Extraction Socket: Integrating Site Phenotype, Pathological Microenvironment, and Healing Sequence

Table S1. Healing events, stage-specific failure risks, and material functional windows in the extraction socket.

Healing events/stages	Major cellular and biological events	Key failure risks	Priority material functions	Functions that should not be enhanced prematurely or excessively	Recommended evaluation indicators	Key references
Hemostasis and blood clot establishment	Platelet activation and fibrin network formation; local enrichment of signals such as PDGF, TGF-β, and VEGF; initiation of leukocyte adhesion and migration	Salivary flushing, local micromotion, enhanced fibrinolysis, and early bacterial colonization	Rapid hemostasis, wet-environment adhesion, washout resistance, blood clot protection, and coronal coverage	High-dose osteogenic factors or strong, sustained anti-inflammatory effects before the wound has stabilized	Clotting time, blood clot retention, material retention, and early pain and exposure	(Languino et al., 1993; Verma et al., 2019; de Sousa Gomes et al., 2019; Udeabor et al., 2023; He et al., 2025)
Inflammatory clearance and transition toward repair	Neutrophils, monocytes, and macrophages clear microorganisms and tissue debris; inflammatory signaling gradually transitions toward a reparative microenvironment	Residual biofilm, persistent pro-inflammatory signaling, impaired macrophage phenotype switching, and premature immunosuppression	Control of the infectious burden, limitation of excessive inflammation, and promotion of timely resolution	Simplifying the “M1→M2” transition as “the faster the better,” or completely eliminating the inflammation required for normal repair	Mature biofilm, inflammatory cytokines, macrophage dynamics, and clearance of necrotic tissue	(McMillan, 1999; Gong et al., 2016; Raziyeva et al., 2021; Li et al., 2023a; Eom et al., 2025; Fonseca et al., 2025; Qi et al., 2025; Saim et al., 2025)

Granulation tissue formation, angiogenesis, and early osteogenesis	Entry of endothelial cells, MSCs, and osteogenic precursors; involvement of BMP/Smad, VEGF, and Wnt-related signals in angiogenesis–osteogenesis coupling	Non-interconnected pores, insufficient blood supply, excessively dense materials, and osteogenic stimulation alone without vascular support	Interconnected pores, cell adhesion, vascular ingrowth, moderate osteoinduction, and temporally sequenced delivery	Burst release of osteogenic signals, excessively strong pro-angiogenic stimulation, or neglect of space maintenance	Vascular density/perfusion, cell migration, osteogenic markers, and new bone formation and maturity	(Nishio et al., 2006; Phimphilai et al., 2006; Towler, 2007; Hu and Olsen, 2016; Komori, 2019; Song et al., 2024a; Ouyang et al., 2025)
Woven bone formation and late-stage remodeling	Coordination among osteoblasts, osteocytes, and osteoclasts; RANKL/OPG participates in bone resorption and the formation of mature lamellar bone	Excessively rapid material degradation leads to loss of space, whereas excessively slow degradation leads to residual particles, foreign body reactions, or delayed bone maturation	Matching of degradation to new bone formation, controllable residual material, and long-term structural stability	Judging efficacy solely on the basis of short-term micro-computed tomography (micro-CT) bone volume	Proportions of new bone/residual material/connective tissue, lamellar bone maturation, and long-term contour stability	(Boyce and Xing, 2008; Dumas et al., 2014; Ghalayani et al., 2014; Rahman et al., 2015; Sadr et al., 2017; Aoyagi et al., 2018; Biguetti et al., 2019; Kim et al., 2020; Bolamperti et al., 2022; Fan et al., 2022; Tajvar et al., 2023; Huang et al., 2025; Kim et al., 2025a)
Cross-species extrapolation	Rodents exhibit rapid metabolism and	Directly equating fixed postoperat	Designing functional windows according	Directly interpreting calvarial, long-bone, or	Completeness of events, model relevance,	(Araújo et al., 2015; Rahman et al., 2015; de Sousa Gomes et al., 2019;

	remodeling; dogs and minipigs more closely resemble human alveolar bone structure but still differ in loading and microbiota	ive days, release periods, and effect sizes in animals with those in humans	to key biological events rather than fixed days; prioritizing validation in direct extraction socket and large-animal models	subcutaneous models as evidence of efficacy in human extraction sockets	and material stability in the open oral environment	Kim et al., 2020; Udeabor et al., 2023; Yin et al., 2025)

Note: The stages overlap with one another; therefore, fixed postoperative days are not used in this table.

Table S2. Mechanism-oriented biomaterial strategies for the extraction socket: functions, applicable sites, directness of evidence, and translational limitations.

**Material category**	**Main functions and engineering mechanisms**	**Priority applicable sites or barriers**	**Representative advantages**	**Key limitations/ risks**	**Directness and sources of existing evidence**	**Representative references**
Collagen sponges, membranes, and hydrogels	Mimics the ECM and supports cell adhesion and migration; used for blood clot protection, coverage, and barriers	Routine intact sites, coronal coverage, and membrane–particle systems	Relatively broad clinical application basis, with good biocompatibility and operability	Weak mechanical properties and rapid degradation in protease-rich environments; cross-linking may reduce cytocompatibility	Direct evidence from human extraction sockets; supplemented by indirect evidence from other intraoral or jawbone models	(Linsenmayer et al., 1978; Zitzmann et al., 1997; Ghanaati, 2012; Toledano et al., 2020; Couso-Queiruga et al., 2021; Xie et al., 2021; Agnes et al., 2025)
Chitosan and its composites	Cationic adhesion, hemostasis, and antibacterial activity; can be	Early hemostasis, wet adhesion, and as an adjunct at sites with	Flexible functional integration, with both antibacterial and gelation potential	Affected by molecular weight, degree of deacetylation, and pH; insufficient	Indirect evidence from other intraoral or jawbone models; supplement	(Fakhri et al., 2020; Confederat et al., 2021; Li et al., 2022; Flores-Espinoza et al., 2023; Elshahat et al., 2024; Miranda et al., 2025)

	combined with collagen, gelatin, or HA	infection risk		direct evidence against oral multispecies biofilms	ed by in vitro mechanistic evidence	
Oxidized cellulose	Fluid absorption, physical packing, and local acidification for hemostasis	Short-term bleeding control and early wound protection	Rapid hemostasis and straightforward clinical operation	Sustained low pH may be unfavorable for osteogenesis and the stability of calcium phosphate materials; does not undertake long-term osteoconduction	Indirect evidence from other intraoral or jawbone models; supplemented by in vitro mechanistic evidence	(Zhang et al., 2020; Li et al., 2023c; Kanagal et al., 2025)
Alginate hydrogels	Ionic cross-linking; mechanical properties and degradation can be adjusted via molecular weight and cross-linking degree	In situ gelation, sequential delivery, and wet-environment coverage	Good processability and delivery capability	Lacks natural cell adhesion sites and usually requires bioactive modification	General bone tissue engineering evidence; supplemented by in vitro mechanistic evidence	(Kong et al., 2004; Ma et al., 2020; Qian et al., 2024; Pintilei et al., 2025)
PRF and autologous blood-derived matrices	Fibrin network carrying platelets, leukocytes, and repair mediators	Blood clot reinforcement and soft tissue repair at intact four-wall sites; auxiliary coverage at complex sites	Autologous origin and rapid preparation; certain clinical support for early pain and soft tissue healing	Large heterogeneity among preparation protocols; limited long-term space maintenance; cannot replace bone	Direct evidence from human extraction sockets	(Pan et al., 2019; Canellas et al., 2020; Castro et al., 2021; Encalada Abad et al., 2023; Siawasch et al., 2025)

				substitute materials			
Conventional bone substitute materials (autologous/allogeneic/xenogeneic/synthetic)	Osteoconduction, volume maintenance, or material replacement at different rates	Thin buccal bone plates, sites requiring contour maintenance, or sites planned for subsequent implant placement	The most mature clinical evidence; can reduce part of the horizontal and vertical dimensional loss	Trade-offs exist among materials in new bone formation, residual particles, and volume stability; no unified "best material"	Direct evidence from human extraction sockets	(Avila-Ortiz et al., 2014; Avila-Ortiz et al., 2019; Majzoub et al., 2019; Canullo et al., 2022; Chisci et al., 2023; Ozzo and Kheirallah, 2024; Madi et al., 2025)
Resorbable and non-resorbable barrier membranes	Restrict the entry of epithelial/non-osteogenic tissue, fix particles, and maintain space	Bone wall defects, membrane–particle systems, and open or primary closure	Can integrate soft tissue competition with regenerative space control	Excessively rapid degradation leads to loss of the barrier function; non-resorbable membranes require removal and increase exposure/infection management	Direct evidence from human extraction sockets; supplemented by indirect evidence from other intraoral or jawbone models	(Zitzmann et al., 1997; Ghanaati, 2012; Barone et al., 2013; Lim et al., 2019; Toledano et al., 2020; Couso-Queiruga et al., 2021; Abtahi et al., 2023)
PLGA/PCL and blended polymers	Molecular weight, ratio, and pore structure modulate mechanics/degradation; drug-loadable	Sites or scaffolds requiring controllable degradation and long-term support	Strong processability, suitable for shaping, drug loading, and 3D fabrication	Acidic degradation of PLGA; slow degradation of PCL; limited evidence from human extraction sockets	Direct evidence from human extraction sockets; supplemented by general bone tissue engineering evidence	(Shive and Anderson, 1997; Gunatillake and Adhikari, 2003; Ostafinska et al., 2015; Hoda et al., 2016; Ivanovski et al., 2024)

HA, β-TCP, and other calcium phosphate materials	Osteoconduction, ion release, and mineralized interface; degradation is influenced by crystalline phase, pores, and sintering	Contour maintenance, osteoconduction, and mineralization of composite scaffolds	Controllable composition, with relatively abundant clinical/animal socket evidence	Slow HA resorption; β-TCP is replaced faster but is affected by manufacturing parameters; radiographically dense areas are not equivalent to functional new bone	Direct evidence from human extraction sockets; supplemented by direct evidence from animal extraction sockets	(LeGeros, 2002; Lu and Zreiqat, 2010; Ho et al., 2016; Jeong et al., 2019; Ozzo and Kheirallah, 2024; Tanvir et al., 2024; Kim et al., 2025b; Sadlik et al., 2025; Saxena et al., 2025)
Polymer–ceramic composite scaffolds	The polymer improves formability and toughness, while the mineral component provides an osteoconductive interface	Bone wall defects and complex sites requiring morphological stability and pore control	Can combine structure, degradation, and biological activity	Component compatibility, batch-to-batch consistency, sterilization, and degradation kinetics are complex	Direct evidence from animal extraction sockets; supplemented by general bone tissue engineering evidence	(Yeo and Kim, 2012; Nie et al., 2015; Dhand et al., 2016; Ho et al., 2016; Farjaminejad et al., 2024; Ma et al., 2024; Hamdy et al., 2025; Kim et al., 2025b)
Antibiofilm /immunomodulatory materials	Chitosan, antimicrobial peptides, Ag/Cu/Zn, or TLR/NF-κB-related modulation; controls microorganisms and regulates inflammation	Infected sites after debridement or persistent inflammation	Can act on residual bacteria and the host response simultaneously	Narrow window between the effective antibacterial concentration and host cell safety; mostly single-strain or indirect models	Indirect evidence from other intraoral or jawbone models; supplemented by general bone tissue engineering evidence and in vitro mechanistic evidence	(Gabriel et al., 2006; Choe et al., 2015; Halkai et al., 2017; Fakhri et al., 2020; Confederat et al., 2021; Li et al., 2022; Soma et al., 2022; Flores-Espinoza et al., 2023; Liang et al., 2023; Elshahat et al., 2024; Holguín-Meráz et al., 2024; Huang et al., 2024; Chen et al., 2025; Miranda et al., 2025; Monje et al., 2025; Shi et al., 2025)

rhBMP-2 and BMP-2/VEGF delivery	Osteoinduction; combined/sequential pro-angiogenic and osteogenic delivery; carriers modulate burst release	Certain complex bone wall defects or sites with insufficient vascular–osteogenic coupling	rhBMP-2 is one of the few bioactive factors with human randomized studies in extraction sockets	Dose/carrier dependence; edema, inflammation, aberrant bone formation, burst release, and cost	Direct evidence from human extraction sockets (rhBMP-2); supplemented by general bone tissue engineering evidence (combined BMP-2/VEGF delivery)	(Young et al., 2009; Zhang et al., 2015; Raina et al., 2016; Wang et al., 2018; Liu et al., 2020; Geng et al., 2021; Onji et al., 2021; Tong et al., 2023; Chernysheva et al., 2025; Khoswanto and Dewi, 2025; Surisaeng et al., 2025)
Programmed/stimuli-responsive hydrogels	Hierarchical or core–shell structures achieve sequential release; pH, ROS, and MMPs trigger dynamic bond cleavage or release	Complex sites with well-defined pathological signals that require staged anti-inflammatory/antioxidant/osteogenic functions	Can theoretically match the temporal sequence and pathological intensity	Real thresholds in humans are unknown; stimuli vary between individuals and across space; sterilization, storage, and degradation products require verification	Direct evidence from animal extraction sockets; supplemented by general bone tissue engineering evidence and in vitro mechanistic evidence	(Elkassih et al., 2019; Dashtimoghadam et al., 2020; Ma et al., 2020; Chen et al., 2021; Lin et al., 2022; Wei et al., 2022; Chen et al., 2023; Li et al., 2023d; Liu et al., 2025a; Liu et al., 2025c; Oh et al., 2025; Zhang et al., 2025; Zheng et al., 2025; Guo et al., 2026; Qiu et al., 2026)
3D printing and individualized scaffolds	CBCT-based morphological adaptation; modulates pores, mechanics, and the spatial distribution	Two-wall/three-wall defects and complex defects in which conventional particles can hardly maintain space	Can improve implantation fit and structural reproducibility	Manufacturing time, cost, sterilization, printing errors, and regulatory burden; net benefit at routine four-wall	Direct evidence from human extraction sockets; supplemented by indirect evidence from other intraoral or	(Goh et al., 2015; Wang et al., 2015; Juodzbalys et al., 2019; Bartnikowski et al., 2020; Dong et al., 2021; Freeman et al., 2021; Anderson et al., 2022; Song et al., 2022; Gao et al., 2023; Jiao et al., 2023; Li et al., 2023b; Ivanovski et

of components		sites is unclear	jawbone models	al., 2024; Ma et al., 2024; Slavin et al., 2024; Müller et al., 2025)

Note: The evidence-directness column reflects the distance of existing evidence from the clinical problem of human extraction sockets, and is not a formal GRADE rating of evidence quality.
